# β-Secretase 1’s Targeting Reduces Hyperphosphorilated Tau, Implying Autophagy Actors in 3xTg-AD Mice

**DOI:** 10.3389/fncel.2015.00498

**Published:** 2016-01-08

**Authors:** Diego Piedrahita, John Fredy Castro-Alvarez, Ryan L. Boudreau, Andres Villegas-Lanau, Kenneth S. Kosik, Juan Carlos Gallego-Gomez, Gloria Patricia Cardona-Gómez

**Affiliations:** ^1^Cellular and Molecular Neurobiology Area, Viral Vector Core and Gene Therapy, University of AntioquiaMedellin, Antioquia, Colombia; ^2^Internal Medicine, University of IowaIowa, IA, USA; ^3^Neurobank, Neuroscience Group of Antioquia, Faculty of Medicine, SIU, University of AntioquiaMedellín, Colombia; ^4^Department of Molecular Cellular Developmental Biology, Neuroscience Research Institute, University of California Santa BarbaraSanta Barbara, CA, USA

**Keywords:** β-secretase 1, chaperones, lipid rafts, tauopathy, autophagy, Alzheimer’s disease

## Abstract

β-site APP cleaving enzyme 1 (BACE1) initiates APP cleavage, which has been reported to be an inducer of tau pathology by altering proteasome functions in Alzheimer’s disease (AD). However, the exact relationship between BACE1 and PHF (Paired Helical Filaments) formation is not clear. In this study, we confirm that BACE1 and Hsc70 are upregulated in the brains of AD patients, and we demonstrate that both proteins show enhanced expression in lipid rafts from AD-affected triple transgenic mouse brains. BACE1 targeting increased Hsc70 levels in the membrane and cytoplasm fractions and downregulated Hsp90 and CHIP in the nucleus in the hippocampi of 3xTg-AD mice. However, these observations occurred in a proteasome-independent manner *in vitro*. The BACE1miR-induced reduction of soluble hyperphosphorylated tau was associated with a decrease in MAPK activity. However, the BACE1 RNAi-mediated reduction of hyperphosphorylated tau was only blocked by 3-MA (3-methyladenine) *in vitro*, and it resulted in the increase of Hsc70 and LAMP2 in lipid rafts from hippocampi of 3xTg-AD mice, and upregulation of survival and homeostasis signaling. In summary, our findings suggest that BACE1 silencing neuroprotects reducing soluble hyperphosphorylated tau, modulating certain autophagy-related proteins in aged 3xTg-AD mice.

## Introduction

“Alzheimer’s disease (AD), the most common cause of senile dementia, is characterized by β-amyloid (βA) plaques, neurofibrillary tangles and extensive neuron loss”. Although the pathogenesis of AD is still controversial, one of the most accepted mechanisms is the β-amyloid hypothesis (Karran et al., 2011; Toyn and Ahlijanian, 2014). BACE1 (β-site APP cleaving enzyme 1) is a transmembrane aspartic protease that is localized to lipid rafts in human brains (Tun et al., 2002; Cordy et al., 2003; Ehehalt et al., 2003) and is upregulated in AD brains (Fukumoto et al., 2002; Ehehalt et al., 2003; Yang et al., 2003; Holsinger et al., 2006; Ahmed et al., 2010). The presence of BACE1 in lipid rafts correlates with cellular stress (Tamagno et al., 2002, 2005, 2008; Tong et al., 2005; Vassar et al., 2009; Oda et al., 2010), ceramides (Cordy et al., 2003; Puglielli et al., 2003; Kalvodova et al., 2005) and plasma and CNS total cholesterol levels (Refolo et al., 2000, 2001; Ehehalt et al., 2003; Ghribi et al., 2006; Grimm et al., 2008; Reed et al., 2014). BACE1-mediated APP processing can occur in endosomes, endoplasmic reticulum/trans-Golgi network or the plasma membrane, but it occurs predominantly in lipid rafts (Riddell et al., 2001; Cordy et al., 2003; Ehehalt et al., 2003; Kins et al., 2006). APP and BACE1 co-segregation may be facilited by changes in local membrane environment during aging, thereby leading to increased β-amyloid production (Cordy et al., 2003; Kins et al., 2006; Paz Gavilan et al., 2006; Vetrivel and Thinakaran, 2006).

Because AD is also considered a misfolding pathology, chaperones play key roles in its etiopathogeny (Balch et al., 2008; Doyle et al., 2013). Hsc70 (HSP73) is an essential “housekeeping” member of the heat shock protein A (HSPA) family that is 86% identical to Hsp70 (Hsp72), with which it shares biochemical and biological characteristics (Kampinga et al., 2009). Hsc70 mediates co-translational folding, protein translocation through intracellular membranes, chaperone-mediated autophagy (CMA), disassembly of clathrin-coated vesicles, and prevents protein aggregation under stress (Cuervo and Dice, 2000a; Massey et al., 2006; Bandyopadhyay et al., 2008). Hsc70 interacts with the Hsc70 interacting protein (CHIP) carboxy terminus, which functions as an intrinsic E3 ubiquitin ligase to promote ubiquitination (Jiang et al., 2001). “CHIP overexpression increases cellular APP levels and promotes both APP and phospho-tau ubiquitination” (Petrucelli et al., 2004; Shimura et al., 2004; Kumar et al., 2007). “*In vitro* binding assays have demonstrated direct interactions between CHIP and both Hsc70 and Hsp70” (Ballinger et al., 1999).

AD patient brains contain significantly higher levels of Hsp70 and Hsc70 (Perez et al., 1991; Lee et al., 2008), and some studies have described the presence of heat shock proteins in lipid rafts (Triantafilou et al., 2002; Broquet et al., 2003). Previous studies have shown that β-amyloid induces tau pathology through direct alterations of proteasome functions (Oddo et al., 2008). Recent reports have strongly implicated Hsp70/Hsp90 in tauopathy, because these chaperones regulate stability and degradation of unfolding protein as, pair helical filaments (PHF); but its hyperphosphorylation state overload the efficiency of proteasome-dependent degradation (Bonini, 2002; Sakahira et al., 2002; Petrucelli et al., 2004; Shimura et al., 2004; Dickey et al., 2007; Luo et al., 2007; Oddo et al., 2008; Jinwal et al., 2011). So, when the refolding or degradation of abnormal tau protein is not executed by the proteasome, autophagy pathways enter in the scene. Also, depending on the phosphorylation state of tau can be degraded by the proteasome and by the autophagy-lysosome system (Ikeda et al., 1998; Murakami et al., 1998; Oyama et al., 1998; Hamano et al., 2008; Wang et al., 2009). In this way, autophagy represents a homeostatic regulatory mechanism to control metabolism and cellular stress-induced protein aggregation (Singh and Cuervo, 2011), by macroautophagy or CMA, which faults under neurodegeneration condition, allowing the hyperphosphorylation of tau and NTFs formation (Villamil-Ortiz and Cardona-Gomez, 2015).

Therefore, since Hsc70 has been previously linked to proteasome and CMA, and its dysfunction to tau pathogenesis. In addition, BACE1 and Hsc70 reside in lipid rafts. Then, in this study, we have focused in to solve the link between BACE1 and tau pathogenesis.

## Experimental Procedures

### Human Brains

Human brains from the Neuroscience Group of Antioquia’s Neurobank (Universidad de Antioquia) were used. A total of ten brains from either sex were evaluated, five brains from patients diagnosed with AD and five control brains from adult patients without a clinical or family history of AD or any other neurodegenerative disease. The brains were optimally preserved by fixing one hemisphere in a buffered solution of 37% formaldehyde and freezing the other hemisphere at −80°C. The inferior temporal gyrus region was dissected from the frozen hemisphere for biochemical analysis, and the fixed brains were sectioned at 1 cm^3^ and sections of 50 μm were obtained for histological, immunohistochemical and immunofluorescence analyses.

### Immunohistochemistry

Human brain sections (50 μm) were pre-treated with 10 Mm Tris, pH 6.0, at 85°C for 5 min. The slices were treated with formic acid (20%) for 20 min to detect the β-amyloid protein. The mouse (50 μm coronal sections) and human brain sections were “treated for 20 min in 0.1 M PB:methanol (1:1) with 1% hydrogen peroxide and then incubated for 1 h in 0.1 M PB with 1% BSA and 0.3% Triton X-100. Slices were then incubated with primary antibodies, mouse anti-human amyloid beta protein (1:250, SIGNET) and rabbit anti-BACE1 C-terminal (485–501; 1:100, Calbiochem), overnight at 4°C in 0.1 M PB with 0.3% BSA and 0.3% Triton X-100. The slices were incubated with a biotinylated mouse secondary antibody and then incubated with ABC-HRP complex (Pierce Biotechnology) for 2 h. Diaminobenzidine (DAB) was used to develop the staining. The tissues were dehydrated, covered with mounting solution and observed on an Eclipse E200 optical microscope (Nikon)”.

### Lipid Raft Isolation

The cerebral cortices and hippocampi were lysed in 1% Triton lysis buffer (25 mM HEPES, pH 6.5, 150 mM NaCl, 2% TX-100, 1 mM EDTA, and 1 mM PMSF) containing a protease inhibitor cocktail. An equal volume of 80% sucrose was transferred to an SW41Ti centrifuge tube and then overlaid with 6.5 ml 30% sucrose solution and 3.5 ml of 5% sucrose solution containing 25 mM HEPES and 150 mM NaCl at pH 6.5. The discontinuous sucrose gradients were ultra-centrifuged for 18 h at 4°C with an SW41Ti rotor (Beckman Instruments, Palo Alto, CA, USA) at 200,000 g. The gradient was then fractionated into 12 fractions from the bottom to the top. Each fraction was then prepared for Western blotting and confirmed with flotillin and clathrin antibodies”.

### RNAi Design

We designed shRNA miR sequences for silencing BACE1 (shRNAmiR-BACE1) using the BACE1 RNAi sequences (version 1.3.) previously published by Kao et al. (2004). Following the same methodologic strategy in Piedrahita et al., 2010, “these sequences were cloned into human miR 30-base stem-loops by polymerase extension of overlapping DNA oligonucleotides. The following primers were used for polymerase extension to clone the RNAi into a lentiviral shuttle plasmid (pCMV-GIN-ZEO.GFP) for transfection in HEK-293T: shBACE1miR forward primer, 5′-CAGAAGGCTCGAGAAGGTATATGCTGTTGACAGTGAGCGCGGACTGCAAGGAGTACAACTATAGTGAAGCCACAGATGTA-3′, and shBACE1miR reverse primer, 5′-CTAAAGTAGCCCCTTGAATTCCGAGGCAGTAGGCATGGACTGCAAGGAGTACAACTATACATCTGTGGCTTCAC-3′. The extension products were digested with XhoI and EcoRI for directional cloning into the pCMV-GIN-ZEO.GFP vector (Open Biosystem). The following primers were used for polymerase extension to clone the RNAi vectors for adeno-associated virus (AAV) production: shBACE1miR forward primer, 5′-AAAACTCGAGGAGCTCGTGAGCGCTGGACTGCAAGGAGTACAACTCTGTGAAGCCACAGATGGG-3′, and shBACE1miR reverse primer, 5′-TTTTGGATCCATTAATAGGCAATGGACTGCAAGGAGTACAACTCCCATCTGTGGCTTCACAG-3′. These extension products were digested with XhoI and SpeI for directional cloning into a U6 expression plasmid that had been digested with XhoI and XbaI (Boudreau et al., 2009)”.

### Viral Particle Production and Neuron Culture Transduction

The protocol for producing “AAV particles for large-scale production of heterologous proteins used Sf9 insect cell culture with co-infection of recombinant baculovirus derived from the *Autographa californica* nuclear polyhedrosis virus (Urabe et al., 2002). The shRNAmir-BACE1 expression cassettes were driven by the mouse U6 promoter and were cloned into pAAV.CMV.hrGFP, which contained AAV serotype 2/5 inverted terminal repeats and a CMV-humanized *Renilla* GFP (hrGFP)-simian virus 40 poly-(A) reporter cassette (Urabe et al., 2002; Boudreau et al., 2009). AAV titers were determined using quantitative PCR and/or DNA slot blot analysis. The AAV particles were dialyzed before use” (Castro-Alvarez et al., 2014).

### Animal Procedures

The animals were housed in the SPF *vivarium* at the SIU-Universidad de Antioquia, Medellín, Colombia. “Animals were handled according to Colombian animal handling regulations (Law 84/1989 and resolution 8430/1993) and NIH animal welfare care guidelines (Public Law 99-158, November 20th, 1985, “Animals in Research”)”. The protocol was approved by “Ethics committee for animal experimentation” from University of Antioquia (September 29-2011).

Neuronal primary cultures were prepared from 10 pregnant Wistar rats at E17-E18. Mice from either sex were used, for a total of 10 C57BL/6 wild-type mice (5–9 months old), 20 18-month-old triple transgenic Alzheimer’s mice (3xTg-AD) that were treated for 6 months and 30 15-month-old 3xTg-AD mice that were treated for 3 weeks.

How previously we have described “The 3xTg-AD mice (Oddo et al., 2003) and wild-type mice were injected with 2 μL of AAV2/5-BACE1miR or AAV2/5-GFP (control) with a titer of 10^12^ genomes per ml into the right hippocampus (Bregma coordinates: −1.7 antero-posterior, −0.7 lateral and −1.75 depth). The injections were performed with a 10 ml Hamilton syringe at a rate of 0.2 μl/min, and 5 min elapsed after the infusion before the syringe was withdrawn. The animals were transcardially perfused with 4% paraformaldehyde in PBS and processed for immunodetection assays. The brains were cryopreserved with 30% sucrose and stored at −20°C. The hippocampi and cerebral cortices were dissected, immediately frozen, placed on dry ice and stored at −80°C until use” (Piedrahita et al., 2010; Gutiérrez-Vargas et al., 2015).

### Western Blotting

Human and mouse brain tissue, neuronal primary cultures and HEK-293T cells were lysed in 150 mM NaCl, 20 mM Tris, pH 7.4, 10% glycerol, 1 mM EDTA, 1% NP40, 100 μM phenylmethylsulfonyl fluoride, 1 μg/ml aprotinin and leupeptin (Sigma), 100 μM orthovanadate (Cardona-Gomez et al., 2004) and a protease inhibitor cocktail. The proteins (30 μg) were loaded on 10% SDS-PAGE gels, separated at 110 V and transferred to nitrocellulose membranes (Amersham) at 250 mA for 2 h using an electrophoretic transfer system. The membranes were incubated overnight at 4°C with rabbit anti-BACE1 C-terminal (485–501; 1:250, Calbiochem), rabbit anti-BACE2 (Ab2; 44–59; 1:500, Calbiochem), rabbit anti-Hsc70 (Hsp73; 1:1000, Assay Designs), mouse anti-Hsp90 (AC88; 1:500, Assay Designs), rabbit anti-CDK5 (C-8; 1:1000, Santa Cruz Biotechnology), mouse anti-human-PHF-tau (1:500, Pierce Biotechnology), rabbit anti-phospho-GSK-3β (Ser9; 1:1000, Cell Signaling Technology), rabbit anti-phospho-Akt (Ser473; 1:1000, Cell Signaling Technology), rabbit anti-CHIP (N-terminal; 1:1000, Sigma-Aldrich), rabbit anti-LC3B (1:500, Cell Signaling Technology), rabbit anti-LAMP2-A (1:1000, Sigma-Aldrich), mouse anti-flotillin-1 (1:1000, BD Biosciences), rabbit anti-mTOR (1:1000, Cell Signaling Technology), mouse anti-Bcl-2 (1:500, Santa Cruz Biotechnology), rabbit anti-HSF1 (1:1000, Cell Signaling Technology), rabbit anti-phospho-mTOR (Ser2448; 1:1000, Cell Signaling Technology), rabbit anti-phospho-p70 S6 kinase (Thr389; 1:1000, Cell Signaling Technology), rabbit anti-beclin-1 (1:1000, Cell Signaling Technology), rabbit anti-FoxO3 (1:1000, Cell Signaling Technology), mouse anti-presenilin-1 (APS 18; 1:250, Pierce Biotechnology), PHF-1 monoclonal antibody, which recognizes TaupSer-396/404 (1:1000) donated by P. Davies (Feinstein Institute for Medical Research, Manhasset, NY, USA), mouse anti-Phospho-PHF-tau pSer202/Thr205 Antibody (AT8; 1:1000, Thermo Fisher Scientific), mouse anti-phospho-PHF-tau pThr212/Ser214 Antibody (AT100; 1:1000, Thermo Fisher Scientific), mouse anti-phospho-PHF-tau pThr231 Antibody (AT180), (1:1000, Thermo Fisher Scientific), mouse anti-Tau Antibody (TAU-5; 1:1000, Invitrogen) anti-APP A4 (Millipore, Billerica, MA 1:500), anti-APP C-terminal antibody (Sigma-Aldrich 1.500), anti-Amyloid β (6E10, signet, Covance 1:1000) and mouse anti-βIII tubulin (1:1000, Promega Corporation) or mouse anti-β Actin (1:2000, Sigma-Aldrich) antibodies. The following secondary antibodies were used: IRDye 800CW goat anti-mouse or rabbit (LI-COR, Inc., diluted 1:5000) and peroxidase-conjugated anti-mouse IgG or anti-rabbit IgG (Jackson Laboratories, diluted 1:10,000) antibodies (Table 1). The blots were developed using an Odyssey Infrared Imaging System or chemiluminescence (ECL Western blotting system, Amersham) followed by exposure to radiographic film (ECL Hyperfilm, Amersham). The films were analyzed using ImageJ Software (NIH) and Quantity One, version 4.3.0 (Bio-Rad)”.

**Table 1:** **Antibodies information**.

Antibody	Laboratory	Dilution
Rabbit–anti-BACE1 C-terminal (485–501)	Calbiochem	1:250
Rabbit anti-BACE2 (Ab2; 44–59)	Calbiochem	1:500
Rabbit anti-Hsc70 (Hsp73)	Assay Designs	1:1000
Mouse anti-Hsp90 (AC88)	Assay Designs	1:500
Rabbit anti-CDK5 (C-8)	Santa Cruz Biotechnology	1:1000
Mouse anti-human-PHF-tau	Pierce Biotechnology	1:500
Mouse anti-presenilin-1 (APS 18)	Pierce Biotechnology	1:250
Rabbit anti-phospho-GSK-3β (Ser9)	Cell Signaling Technology	1:1000
Rabbit anti-phospho-Akt (Ser473)	Cell Signaling Technology	1:1000
Rabbit anti-CHIP (N-terminal)	Sigma-Aldrich	1:1000
Rabbit anti-LC3B	Cell Signaling Technology	1:500
Rabbit anti-LAMP2-A	Sigma-Aldrich	1:1000
Mouse anti-flotillin-1	BD Biosciences	1:1000
Rabbit anti-mTOR	Cell Signaling Technology	1:1000
Rabbit anit-phospho-mTOR (Ser2448)	Cell Signaling Technology	1:1000
Mouse anti-Bcl-2	Santa Cruz Biotechnology	1:500
Rabbit anti-HSF1	Cell Signaling Technology	1:1000
Rabbit anit-phospho-p70 S6	Cell Signaling Technology	1:1000
kinase (Thr389)
Rabbit anit-p70 S6 kinase	Cell Signaling Technology	1:1000
Rabbit anti-beclin-1	Cell Signaling Technology	1:1000
Rabbit anti-FoxO3	Cell Signaling Technology	1:1000
Mouse anti-Phospho-PHF-tau pSer202 +	Thermo Fisher Scientific	1:1000
Thr205 Antibody (AT8)
Mouse anti-phospho-PHF-tau pThr212 +	Thermo Fisher Scientific	1:1000
Ser214 Antibody (AT100)
Mouse anti-phospho-PHF-tau pThr231	Thermo Fisher Scientific	1:1000
Antibody (AT180)
Mouse anti-Tau Antibody (TAU-5)	Thermo Fisher Scientific	1:1000
Rabbit anti-APP A4	Millipore, Billerica, MA	1:500
Rabbit anti-APP C-terminal antibody	Sigma-Aldrich	1:500
Mouse anti-Amyloid *β* (6E10)	Signet, covance	1:1000
Mouse anti-βIII tubulin	Promega Corporation	1:1000
Mouse anti-β Actin	Sigma-Aldrich	1:2000
IRDye 800CW goat anti-mouse or rabbit	LI-COR, Inc.	1:5000
Peroxidase-conjugated anti-mouse IgG	Jackson Laboratories	1:10,000
Peroxidase-conjugated anti-rabbit IgG	Jackson Laboratories	1:10,000

### Immunofluorescence Microscopy

“The mouse brains were cut into 50 μm coronal sections with a vibratome (Leica 1000) and treated with 50 mM ammonium chloride (NH_4_Cl) for 10 min at room temperature. The slices were pre-incubated for 1 h in 1% BSA with 0.3% Triton X-100 in 0.1 M PB. The primary antibodies were incubated overnight at 4°C: rabbit anti-BACE1 C-Terminal (485–501; 1:250, Calbiochem), rabbit anti-BACE2 (Ab2; 44–59; 1:250, Calbiochem), mouse anti-human-PHF-tau (1:250, Pierce Biotechnology), and rabbit anti-Hsc70 (Hsp73; 1:250, Assay Designs). Alexa 488- and 594-conjugated secondary antibodies (Molecular Probes) were used. The slices were observed by fluorescence microscopy (Olympus IX81), and the individual images for GFP, BACE1, BACE2, PHF and Hsc70 expression were analyzed using Image Scope Pro software (Media Cybernetics). Deconvolution was performed using Image Scope Pro software (Media Cybernetics) and Cell Software (Olympus)”.

### Measuring Aβ40 and Aβ42 Levels

“The Aβ40 and Aβ42 protein levels from the hippocampi of 15-month-old 3xTg-AD mice that had been treated with AAV2/5-BACE1miR (BACE1miR) or AAV2/5-GFP (GFP) for one month were measured by ELISA, as described in the manufacturer’s instructions (BetaMark x-42 ELISA Protocol-SIG-38956-kit and BetaMark x-40 ELISA Protocol-SIG-38950-kit)”.

### Soluble and Insoluble Tau Quantification

The hippocampi from 15-month-old 3xTg-AD mice that had been treated with AAV2/5-BACE1miR (BACE1miR) or AAV2/5-GFP (GFP) for three weeks were lysed in “150 mM NaCl, 20 mM Tris, pH 7.4, 10% glycerol, 1 mM EDTA, 1% NP40, 100 μM phenylmethylsulfonyl fluoride, 1 μg/ml aprotinin and leupeptin (Sigma), 100 μM orthovanadate (Cardona-Gomez et al., 2004) and a protease inhibitor cocktail. The lysates were centrifuged at 13,000 rpm at 4°C for 10 min. A fraction of the supernatant was stored as the soluble fraction. The remaining fraction was diluted in sarkosyl buffer (50 mM Tris HCl, pH 7.4; 0.15 M NaCl, 1% lauryl sarcosamine, and protease inhibitor cocktail) and centrifuged at 13,000 rpm at 4°C for 10 min. The supernatant was incubated for 30 min at RT and centrifuged at 170,000 g for 2 h. The pellet was diluted in sarkosyl buffer and stored as the insoluble fraction. The soluble and insoluble fractions were analyzed by Western blotting, as described above”.

### *In Vitro* CDK5 Kinase Assay

“Neuronal primary cultures were transduced for 7 days with AAV2/5-BACE1miR or AAV2/5-GFP (transduction control) and then placed in 1.5 ml microfuge tubes containing lysis buffer, rapidly frozen using liquid nitrogen immersion, and kept frozen until the assay was performed. Then, the sample was thawed on ice, homogenized, incubated for 15 min on ice, and centrifuged at 13,000 rpm at 4°C. The supernatant was recovered in clean microfuge tubes, and the protein concentration was measured with the bicinchonic acid method (Thermo Fisher Scientific). CDK5 was immunoprecipitated from 250 μg of total protein using 1 μg of the rabbit polyclonal anti-CDK5 (C-8) antibody (Santa Cruz). The antibody was incubated with the protein extract overnight at 4°C on a rotator. Protein G-Sepharose (Sigma-Aldrich) was added, and the samples were incubated for an additional 1 h at 23°C. The Protein G-Sepharose beads were washed five times with immunoprecipitation (IP) buffer (Sigma-Aldrich), while maintaining the sample at 4°C. After the fifth wash, the Protein G-Sepharose beads were resuspended in 200 μl of kinase assay buffer (20 mM Tris-HCl, pH 7.5, 100 μM sodium orthovanadate, 10 mM MgCl_2_, 50 mM NaCl, 1 mM DTT, and 1 mM NaF), and ATP was added to the resuspended beads at a 10-fold excess (0.5 mM). Histones from calf thymus type III-S (Sigma- Aldrich) were added at a final concentration of 6 μM as a substrate for CDK5, and then the reaction was gently vortexed, aliquoted and incubated at 37°C for 30 min. To stop the reaction, 5 μl of SDS-PAGE loading buffer (250 mM Tris-HCl, 10% SDS, 30% glycerol, 0.5 M DTT, 0.02% bromophenol blue) was added, and the samples were immediately incubated for 5 min at 95°C. The samples were separated electrophoretically at 120 V for 2 h and transferred to a nitrocellulose membrane at 200 mA for 1.5 h. Ponceau Red in 5% acetic acid was used to stain the transferred proteins. The histones were clearly visible, and migrated at approximately 21 kDa on the gel. Western blots for CDK5 (C-8 antibody) and rabbit polyclonal anti-phosphorylated histone H1 (Millipore; 06-597) were used. Goat anti-rabbit IRDye 800WE (LI-COR) was used as the secondary antibody and detected using an Odyssey Infrared Imaging System (LI-COR). The band intensities for the histones were measured with NIH ImageJ software and normalized to the IgG heavy chain intensity”.

### *In Vitro* MAP Kinase Assay

We used the MAP Kinase/Erk IP Kinase Assay kit (Millipore # 17-192) according to the manufacturer’s instructions. “The assay kit is designed to measure phosphotransferase activity in an immunocomplex formed between the MAP Kinase R2 antibody and MAP Kinase (p44^mpk^). This precipitated enzyme is used to phosphorylate a specific substrate, myelin basic protein (MBP). The phosphorylated substrate is then analyzed by Western blotting using an antibody specific for phosphorylated MBP. The measurement of MAPK activity in most cell lysates is not accurate due to the phosphorylation of MBP by other kinases”.

### PP2A Activity Measurement

“PP2A phosphatase activity from the hippocampi of 3xTg-AD mice (18-month-old) treated for 6 months with AAV2/5-BACE1miR or AAV2/5-GFP (control), were analyzed with a PP2A IP Phosphatase Assay Kit (Millipore) as described in the manufacturer’s instructions”.

#### Cellular Fractions

The cerebral cortices and hippocampi from wild-type and 3xTg-AD mice were “homogenized in 100 mM Tris, pH 7.4, 3 mM MgCl_2_, 0.32 M sucrose, 0.1% Triton X-100 (Buffer A), and a protease inhibitor cocktail (Sigma-Aldrich). The soluble fraction was obtained after centrifuging at 2500 rpm for 15 min at 4°C. The pellet was suspended in Buffer A and loaded on a two-layer sucrose cushion; the first one was Buffer B containing 1.9 M sucrose and the second, Buffer C, contained 2 M sucrose. The sample was then centrifuged for 1 h at 10,000 rpm at 4°C. The membrane fraction was at the top of the 2 M sucrose layer, whereas the nuclear fraction was at the bottom”.

#### *In Vitro* Assays

“Cortical primary cultures (5 × 10^5^ cells/well or 1 × 10^6^ cells/well) from C57BL/6 mice or Wistar rat embryos (E17-E18) were dissected, trypsinized, dissociated and cultured on poly-L-lysine-coated (Sigma-Aldrich) 24-well or 6-well plates respectively, in Neurobasal medium (GIBCO) containing B-27 supplement, (Sigma-Aldrich), and penicillin-streptomycin (GIBCO), at 37°C in a 5% CO_2_ humidified atmosphere. At DIV5, the neuronal primary cultures in 6-well plates were transduced with 2 μl of AAV2/5-BACE1miR or AAV2/5-GFP (transduction control) with 10^12^ genomes per ml titer for 7 days” (Piedrahita et al., 2010). At DIV 12, the neurons were exposed to lactacystin (synthetic; 10 μM, Calbiochem), KNK437 heat shock protein inhibitor I, (100 μM, Calbiochem), or the autophagy inhibitors 3-methyladenine (10 mM, Sigma-Aldrich), bafilomycin (100 nM, Sigma-Aldrich), ammonium chloride (NH4Cl, 20 mM, Sigma-Aldrich) or DMSO (Sigma-Aldrich) for 24 h.

#### Statistical Analysis

“The n used for *in vitro* and *in vivo* experiments were 3–6. Parametric data were compared using multi-variable two-way analysis of variance (ANOVA) followed by Tukey’s *post hoc* test for comparisons between several independent groups. A *p* < 0.05 confidence level using a two-tailed test was adopted as statistically significant. The Student’s *t*-test was used to compare two groups. The data were expressed as the means ± SEM. The analyses were performed with SPSS (IBM) and GraphPad Prism version 4.00, 2003 (GraphPad Software Inc., San Diego, CA, USA)”.

## Results

### BACE1 and Hsc70 were Upregulated in AD Brains, and Hsc70 is Retained in Lipid Rafts

**Figure 1:**
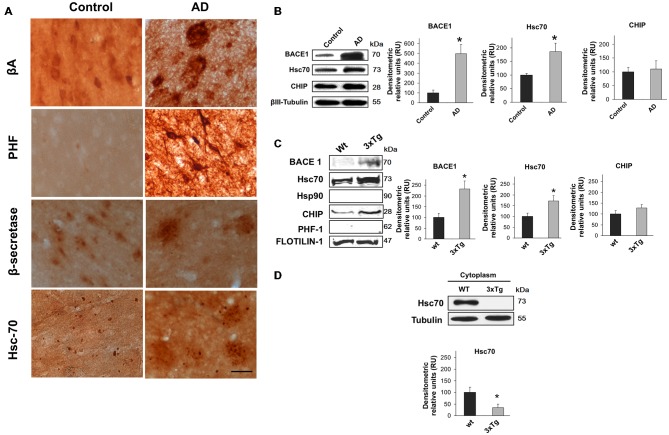
**BACE1 and Hsc70 in AD-affected human brains and in lipid rafts from triple-transgenic Alzheimer’s disease mouse brains. (A)** β-amyloid, PHF-1, BACE1 and Hsc70 immunoreactivity in the temporal gyrus of AD-affected brains (AD) and control brains. 40× magnification; scale bar: 20 μm. *n* = 4. **(B)** BACE1, Hsc70 and CHIP Western blotting from the temporal gyrus of AD brains (AD); normal human brains were used as controls. A representative plot is shown. βIII-tubulin was used as a loading control. Densitometric quantification was performed; RU = relative units. *n* = 4, *p < 0.05. **(C)** Western blotting of BACE1, Hsc70, Hsp90, CHIP and PHF-1 proteins in lipid rafts isolated from the cerebral cortex of 15-month-old wild-type and 3xTg-AD mice. Flotillin-1 was used as a loading control. Densitometric quantification was performed; RU = relative units. *n* = 3, *p < 0.05. **(D)** Western blotting of Hsc70 in the cytoplasmic fraction from the cerebral cortex of wild-type and 3xTg-AD mice. RU = relative units. *n* = 3, *p < 0.05. Representative blots are shown. The arrow shows the band corresponding to the expected molecular weight of the BACE1 protein.

The relationship between BACE1 and Hsc70 was evaluated in the inferior temporal gyrus of AD-affected human brains. The typical hallmarks of AD (β-amyloid and PHF-1) were detected, and BACE1 and Hsc70 immunoreactivity were increased (Figure 1A). BACE1 and Hsc70 protein levels were upregulated, whereas a related protein, the CHIP carboxyl terminus, remained unchanged compared to the levels in control brains (Figure 1B).

It is widely accepted that BACE1 is enriched in lipid rafts in AD (Riddell et al., 2001; Cordy et al., 2003; Ehehalt et al., 2003). Lipid rafts were isolated from 15-month-old triple transgenic mouse brains (3xTg-AD) to determine whether BACE1 and Hsc70 were associated in these micro-domains. BACE1 and Hsc70 proteins were significantly increased in isolated lipid rafts (flotillin-positive fractions, data not shown). CHIP was not changed and Hsp90 and PHF-1 proteins were not detected (Figure 1C). Interestingly, Hsc70 levels were significantly reduced in the cytoplasmic fractions from the brains of the 3xTg-AD mice in comparison with the wild-type mice (Figure 1D). These data suggest that the additional Hsc70 is retained in lipid rafts when BACE1 is increased in this micro-domain in AD brains.

### Specific Silencing of BACE 1 Reduces β-Amyloidosis in the Hippocampus of 3xTg-AD Mice

**Figure 2:**
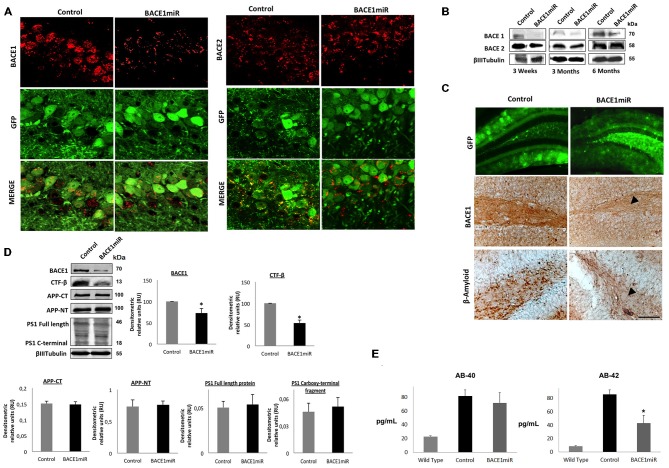
**BACE1miR produces specific BACE1 silencing and reduces β-amyloidosis in 3xTg-AD mice. (A)** Representative confocal images of BACE1 and BACE2 immunofluorescence in the hippocampus from wild-type mice at 6 months after injection with AAV2/5-BACE1miR compared to mice with AAV2/5-GFP injection (Control). Green: GFP fluorescence, red: Alexa 594. 60× magnification; scale bar: 20 μm. *n* = 3. **(B)** Representative immunoblots showing BACE1 and BACE2 protein levels from the hippocampi of wild-type mice at 3 weeks, 3 months and 6 months after injection with AAV2/5-BACE1miR compared to the control values (AAV2/5-GFP). *n* = 3. **(C)** β-secretase 1 (BACE1) and β-amyloid immunoreactivities in the dentate gyrus (DG) of 18-month-old 3xTg-AD mice treated with AAV2/5-BACE1miR or AAV2/5-GFP as control for 6 months. The black arrowheads show the downregulation of the BACE1 and β-A immunostaining. Green: GFP fluorescence, adeno-associated virus (AAV) distribution in the DG. 40×, scale bar: 20 μm. *n* = 3. **(D)** Representative immunoblots showing BACE1, the C-terminal fragment (CTF-β), APP-CT, APP-NT, full-length PS1 and the C-terminal fragment of PS1 from the hippocampi of 18-month-old 3xTg-AD mice evaluated at 6 months after injection of AAV2/5-BACE1miR or AAV2/5-GFP as a control. RU = relative units. *n* = 4, *p < 0.05. **(E)** β-amyloid 1–40 and 1–42 levels from the hippocampi of 18-month-old 3xTg-AD mice were evaluated by ELISA at 6 months after the injection of AAV2/5-BACE1miR or AAV2/5-GFP; C57BL/6 mice were also used as controls. Absorbance was measured at 620 nm. *n* = 5, *p < 0.05.

We generated a recombinant AAV (serotype 2/5), which expresses BACE1miR in addition to a GFP reporter. We evaluated the effect of the BACE1 shRNA-miR treatment on β-amyloidosis in 3xTg-AD mice. Initially, the AAV:BACE1miR vector was injected into the right hippocampus of 5–9-month-old wild-type mice to evaluate gene silencing at 3 weeks, 3 months and 6 months post-injection. We observed reduced BACE1 protein levels in the brains that were injected with the BACE1miR compared to the GFP control as detected by Western blotting (Figure 2A) and confocal immunofluorescence analysis; BACE2 expression was not affected (Figure 2B). In addition, there was a significant decrease in BACE1 as well as β-amyloid immunoreactivity in the 3xTg-AD mice at 6 months after injection with the BACE1miR compared to the GFP-treated animals (Figure 2C). We also confirmed the reduced levels of BACE1 by Western blotting. Moreover, the level of the CTF-β fragment was significantly reduced; however, the levels of APP-CT, APP-NT, full-length PS1 and C-terminal PS1 proteins did not change (Figure 2D). BACE1miR specifically reduced Aβ-42 levels, without changing the Aβ-40 levels (Figure 2E). Therefore, our findings confirm that the BACE1miR reduced BACE1 levels and β-amyloidosis, as previously reported (Luo et al., 2001; Kao et al., 2004; Ohno et al., 2004; Laird et al., 2005).

### BACE1 Targeting Decreases Soluble Hyperphosphorylated Tau by Reducing MAP Kinase Activity in the Hippocampus of 3xTg-AD Mice

**Figure 3:**
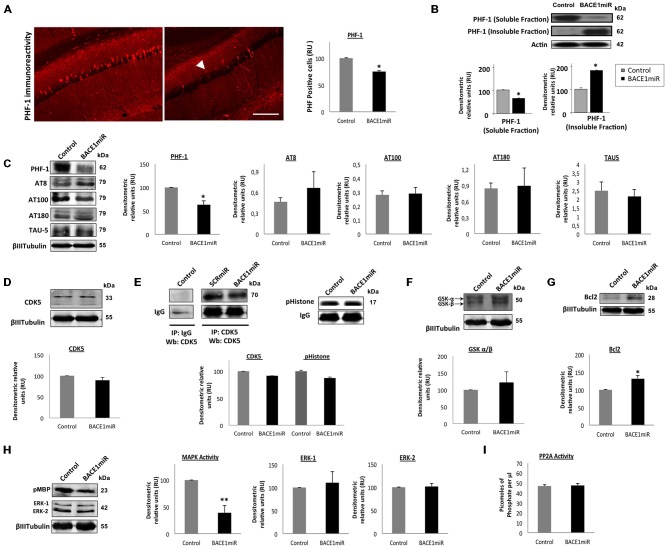
**BACE1miR reverses hyper-phosphorylated tau in the hippocampus of 3xTg-AD mice. (A)** The number of PHF-positive cells in the CA1 region of the hippocampus of 18-month-old 3xTg-AD mice that were treated with AAV2/5-BACE1miR or AAV2/5-GFP as control for 6 months. The white arrowhead shows a significant decrease in immunoreactivity. Representative confocal-DSU images ofimmunofluorescence are shown. Red: Alexa 594, 10× magnification, scale bar: 20 μm. *n* = 3, *p < 0.05. **(B)** Representative bands of the soluble and insoluble fractions of tau. *n* = 3, *p < 0.05. **(C)** Levels of the hyperphosphorylated tau (PHF-1, AT8, AT100, AT180 and TAU-5) protein from the hippocampi of 18-month-old 3xTg-AD mice at 6 months after injection with AAV2/5-BACE1miR or AAV2/5-GFP as control. Representative blots are shown. RU = relative units. *n* = 4, *p < 0.05. **(D)** CDK5, **(E)** CDK5 activity, **(F)** GSK3- β, **(G)** Bcl-2, **(H)** MAPK activity and ERK-1/ERK-2 protein levels were detected. **(I)** Protein phosphatase 2 (PP2A) activity was quantified using the PP2A Immunoprecipitation (IP) Phosphatase Assay Kit (Millipore), which detects picomoles of phosphate per microliter. *n* = 4, *p < 0.05. Representative blots are shown. RU = relative units. *n* = 4, *p < 0.05, **p < 0.001.

Unexpectedly, a significant reduction in the number of PHF-positive cells was observed in the hippocampus (Figure 3A). However, only the level of soluble tau was reduced by BACE1miR, and the level of insoluble tau was even increased (Figure 3B). In addition, PHF-1 protein levels were reduced by BACEmiR, whereas the levels of AT-8, AT-100, AT-180 and TAU-5 were not changed (Figure 3C). To understand the cellular mechanism of hyperphosphorylated tau reduction, we evaluated the various kinases involved, as well as protein phosphatase 2A (PP2A) and Bcl-2. The CDK5 protein levels (Figure 3D), CDK5 activity (Figure 3E), GSK-β, GSK-β (Figure 3F), Bcl-2 (Figure 3G), ERK-1, ERK-2 (Figure 3H) and PP2A activity (Figure 3I) were not modified in BACE1miR-treated 3xTg-AD mice. Interestingly, our data showed that MAPK activity was significantly reduced by BACE miR (Figure 3H), while Bcl-2 was upregulated (Figure 3G), suggesting impacts on cell survival.

### BACE1 Silencing Upregulated Hsc70 in the Cytoplasm and Reduced Hsp90 in the Nucleus

**Figure 4:**
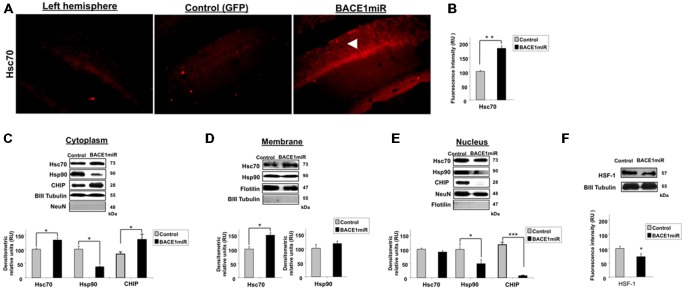
**BACE1miR decreases HSF-1, upregulates Hsc70 and induces differential cellular distribution of Hsc70 and Hsp90 in the hippocampus of 3xTg-AD mice. (A)** Hippocampi of 18-month-old 3xTg-AD mice at 12 months after injection with AAV2/5-BACE1miR (BACE1miR) or AAV2/5-GFP (GFP). Immunofluorescence of Hsc70 in the CA1 region of 3xTg-AD mice. The white arrowheads show a significant increase in Hsc70 protein expression in the CA1 region that was injected with AAV2/5.BACE1miR compared to the left hemisphere (not injected) and to a right hemisphere injected with AAV2/5-GFP (control). Red: Alexa 594. 10X magnification, scale bar: 20 μm. *n* = 3. **(B)** Graph of Hsc70 fluorescence intensity (red channel) in the CA1 area showing a significant increase of Hsc70 expression in BACE1miR-treated mice compared to the left hemisphere (not injected) and right hemisphere injected with AAV2/5-GFP (control). Quantification was performed using image Scope Pro software (Media Cybernetics); RU = relative units. *n* = 3, *p < 0.05. **(C)** Cellular fractions from the hippocampi of 15-month-old 3xTg-AD mice that were treated with AAV2/5-BACE1miR (BACE1miR) or AAV2/5-GFP (GFP) for three weeks. Hsc70, CHIP and Hsp90 levels in the **(C)** cytoplasm, **(D)** membrane and **(E)** nuclear fractions. **(F)** HSF-1 protein expression in the hippocampi of 15-month-old 3xTg-AD mice at three weeks after injection with AAV2/5-BACE1miR (BACE1miR) or AAV2/5-GFP (GFP). Representative blots are shown. βIII tubulin, flotillin and NeuN were used as loading controls or as fraction controls. Densitometric quantification was performed; RU = relative units. *n* = 6, *p < 0.05, **p < 0.001, ***p < 0.0001.

To examine the relationship between BACE1 and Hsc70, Hsc70 protein expression was evaluated in the brains of BACE1miR-treated 3xTg-AD mice. The brain slices showed a significant increase in Hsc70 fluorescence intensity in the CA1 area compared to the GFP control group (Figures 4A,B). The subcellular localization of Hsc70 is critical for its specific functions (D’Souza and Brown, 1998; Chen and Brown, 2007). The localization of Hsc70 and of other related proteins was determined by cellular fractionation and Western blotting from the hippocampi of BACE1miR-treated 3xTg-AD mice. Hsc70 was significantly increased in the cytoplasm and membrane fractions; however, its expression in the nucleus was not changed. Interestingly, the Hsp90 protein level was decreased in the cytoplasm and nuclear fractions, but it remained unchanged in the membrane fraction. Moreover, CHIP was significantly increased in the cytoplasm and decreased in the nucleus (Figures 4C–E). Also, HSF-1 was reduced by the BACEmiR treatment, meaning a non transcriptional upregulation of Hsc 70 (Figure 4F).

### BACE1miR Reduced Hyperphosphorylated Tau in a Proteasome-Independent Manner *In Vitro*

**Figure 5:**
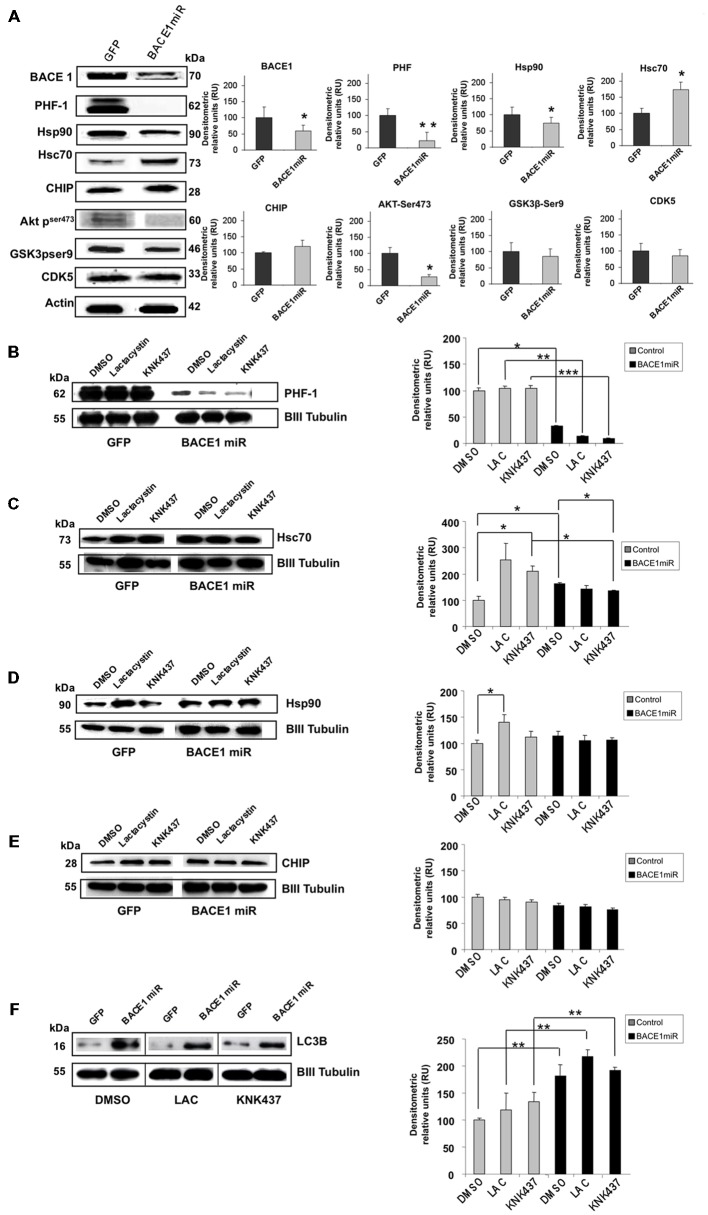
**BACE1miR decreased hyper-phosphorylated tau in a proteosome-independent manner in primary neuronal cultures. (A)** BACE1, PHF-1, Hsp90, Hsc70, CHIP, pAkt (ser473), GSK3 (pSer9) and CDK5 Western blots from primary neuronal cultures transduced at DIV5 (7 days transduction) with AAV2/5-BACE1miR or AAV2/5-GFP as a control. Representative blots are shown. Actin was used as a loading control. Densitometric quantification was performed; RU = relative units. *n* = 4, *p < 0.05, *p < 0.001. **(B)** Western blotting of primary cortical cultures transduced with AAV2/5-BACE1miR or AAV2/5-GFP at DIV5 for 7 days. Neurons were exposed to lactacystin (synthetic; 10 μM), KNK437 heat shock protein inhibitor I, (100 μM) or DMSO for 24 h. Representative blots of **(B)** PHF-1, **(C)** Hsc70, **(D)** Hsp90, **(E)** CHIP and **(F)** LC3-B. βIII-tubulin was used as a loading control. Densitometric quantification was performed; RU = relative units. *n* = 4, *p < 0.05, **p < 0.001, ***p < 0.0001.

As in the *in vivo* model, using protein extracts from transduced cultured cortical neurons, we confirmed that AAV2/5-BACE1miR downregulated BACE1, PHF-1, and Hsp90 and upregulated Hsc 70, while CHIP remained unchanged (Figure 5A). BACE1miR did not affect the typical enzymes involved in tau hyperphosphorylation, such as GSK-3 and CDK5; however, pSer473 Akt showed a modest decrease (Figure 5A). We used these primary neuronal cultures and the proteasome inhibitor lactacystin to assess whether PHF clearance by BACE1 silencing was proteasome-dependent.

Neuronal cultures transduced with AAV2/5-BACE1miR maintained a significant reduction in PHF-1 protein levels despite treatment with lactacystin (proteasome inhibitor) and KNK437 (heat shock protein inhibitor; Figure 5B). As expected, lactacystin increased the levels of Hsp90 and Hsc70, whereas cells treated with BACE1miR did not show increases in Hsp90 and Hsc70. KNK437 produced similar effects. CHIP was not modified by any treatment (Figures 5C–E).

### The BACE1miR-Mediated Reduction in Hyperphosphorylated Tau was Blocked by 3-MA, an Autophagy Inhibitor

In addition to the proteasome pathway, macroautophagy is another important mechanism that is involved in the unfolded protein response and is specifically related to aggregated tau (Ikeda et al., 1998; Murakami et al., 1998; Wang et al., 2006a, 2009; Hamano et al., 2008). Dysfunction of this pathway has been reported in AD (Cataldo et al., 2004a,b; Yu et al., 2004, 2005; Nixon et al., 2005; Boland et al., 2008). A macroautophagy marker, LC3B, was evaluated in the *in vitro* experiment. We found that LC3B was significantly increased in BACE1miR-treated neuronal primary cultures, and this increase was not reversed by lactacystin or KNK437 (Figure 5F). Together, these data suggest a proteasome-independent mechanism for hyperphosphorylation tau loss induced by BACE1miR that involves the up-regulation of LC3B.

**Figure 6:**
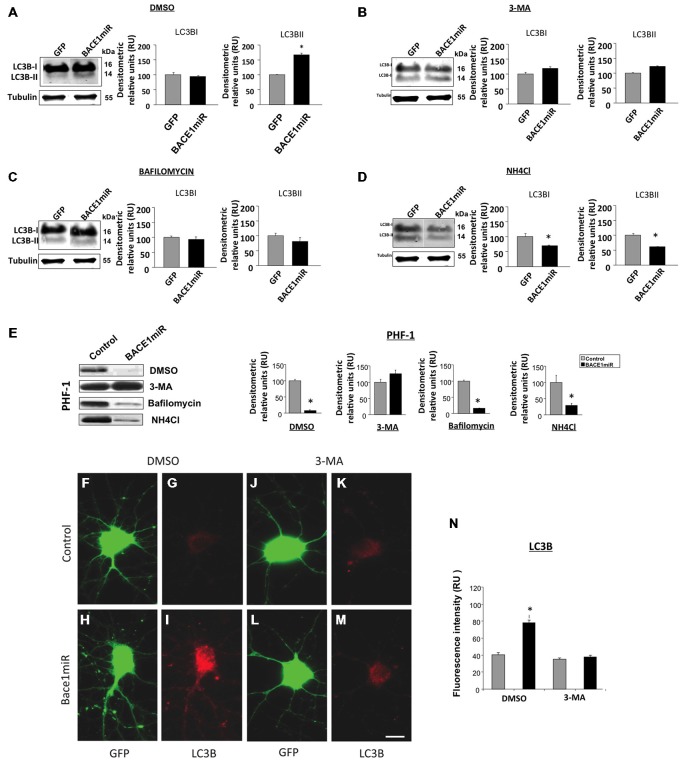
**BACE1miR reduces the level of hyperphosphorylated tau, and this effect is blocked by 3-MA.** Primary cortical cultures were transduced with AAV2/5-BACE1miR or AAV2/5-GFP at DIV5 for 7 days. The neurons were exposed to autophagy inhibitors, including 3-methyladenine (10 mM), bafilomycin (100 nM), ammonium chloride (NH_4_Cl, 20 mM) or DMSO for 24 h. **(A)** Representative bands and quantification of the levels of LC-3B I and II proteins from neurons transduced with AAV2/5-BACE1miR or AAV2/5-GFP and treated with DMSO, **(B)** 3-methyladenine (10 mM), **(C)** bafilomycin (100 nM), and **(D)** ammonium chloride (NH_4_Cl, 20 mM). RU = relative units, *n* = 3, *p < 0.05.** (E)** Representative Western blots for PHF-1 from neurons transduced with AAV2/5-GFP or AAV2/5-BACE1miR. βIII-tubulin was used as a loading control. Densitometric quantification was performed; RU = relative units. *n* = 3, *p < 0.05. **(F,J)** GFP expression in hippocampal neurons transduced with AAV2/5-GFP (control). **(H,L)** GFP expression in hippocampal neurons transduced with AAV2/5-BACE1miR (BACE1miR). **(G,K)** LC3B immunofluorescence in neurons transduced with AAV2/5-GFP (control). **(I,M)** LC3B immunofluorescence in neurons transduced with AAV2/5-BACE1miR (BACE1miR). **(F–I)** Neurons were treated with DMSO as a control; **(J–M)** neurons were treated with 3-methyladenine (10 mM). Green: GFP fluorescence, red: Alexa 594 fluorescence. 60X magnification; scale bar: 20 μm. *n* = 3. **(N)** Quantification of the LC3B fluorescence intensity using Scope-Pro image software (Media Cybernetics) in neurons transduced with AAV2/5-BACE1miR or AAV2/5-GFP and treated with 3-methyladenine (10 mM) or DMSO; RU = relative units, *n* = 3, *p < 0.05.

Primary neuronal cultures transduced with AAV2/5-BACE1miR or AAV2/5-GFP were exposed to autophagy inhibitors, including 3-methyladenine (3-MA, 10 mM, macroautophagy inhibitor), bafilomycin (100 nM, autophagolysosome inhibitor), ammonium chloride (NH_4_Cl, 20 mM, lysosome inhibitor), or DMSO for 24 h to analyze the involvement of the autophagic pathway in the BACE1 silencing effects on PHF immunoreactivity. As expected, LC3B-II (autophagosome formation marker) was significantly upregulated by BACE1miR (Figure 6A). Likewise, 3-MA (Figure 6B), bafilomycin (Figure 6C) and NH_4_Cl (Figure 6D) reversed the effects of BACE1miR on LC3B-II, while LC3B-I was only decreased by NH_4_Cl treatment (Figure 6D). However, the BACE1miR-mediated reduction of PHF-1 protein levels was blocked only by the inhibitor 3-methyladenine and not by bafilomycin or ammonium chloride (Figure 6E). Quantification of the LC3B fluorescence intensity (Figures 6F–N) confirmed that BACE1miR increased LC3B-II, which was blocked by 3-MA. Therefore, these results suggest that phosphatidylethanolamine lipidation is necessary for the BACE1 silencing-induced dephosphorylation of tau, supported by the increased LC3B immunoreactivity, but maybe for a non conventional pathway.

### BACE1 Targeting Induces Hsc70/LAMP2 Upregulation in Lipid Rafts from Hippocampi of 3xTg-AD Mice

**Figure 7:**
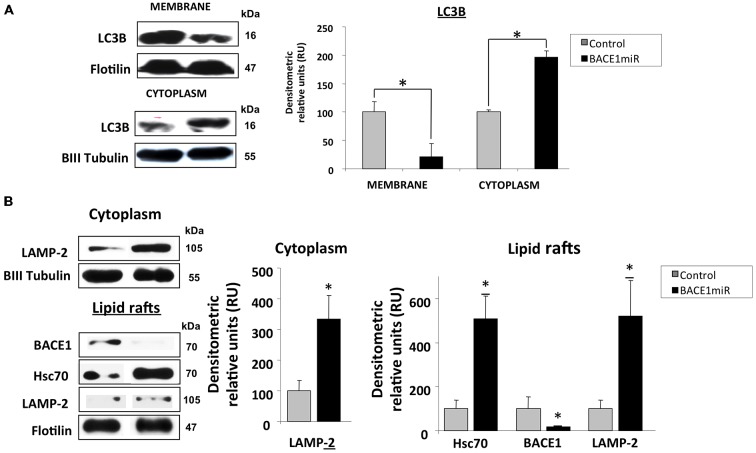
**BACE1 silencing upregulates Hsc70 and LAMP-2.** Hippocampi of 15-month-old 3xTg-AD mice were analyzed at three weeks after injection with AAV2/5-BACE1miR (BACE1miR) or AAV2/5-GFP (GFP). **(A)** LC3-B protein levels in the membrane and cytoplasm fractions. **(B)** BACE1, Hsc70, and LAMP-2A protein levels in lipid rafts and LAMP2-A in the cytoplasm from hippocampi of 15-month-old 3xTg-AD mice treated for three weeks with AAV2/5-BACE1miR (BACE1miR) or AAV2/5-GFP (GFP). βIII tubulin and flotillin were used as a loading control and a fraction control respectively Densitometric quantification was performed; RU = relative units. *n* = 4, *p < 0.05.

The LC3B protein was significantly upregulated in the cytoplasm and decreased in membrane fractions (Figure 7A) from the hippocampi of 3xTg-AD mice treated with BACE1miR compared to the untreated mice.

Both microautophagy and CMA processing require the participation of Hsc70/Hsp90, and these proteins were clearly modified by BACE1miR in a proteasome-independent manner. Therefore, we evaluated whether LAMP-2A, a CMA-induced lysosome membrane receptor (Agarraberes et al., 1997; Cuervo and Dice, 2000a,b; Tanaka et al., 2000; Bampton et al., 2005; Kaushik et al., 2006; Kiffin et al., 2007; Bandyopadhyay et al., 2008), was also modulated by BACE1 knockdown in 3xTg-AD mice. Surprisingly, we found that BACE1miR significantly increased the levels of LAMP-2A and Hsc70 in lipid rafts and in the cytoplasmic fraction from 3xTg-AD hippocampi (Figure 7B).

### BACE 1 Knockdown Induces Survival Signaling in the Hippocampus of 3xTg-AD Mice

**Figure 8:**
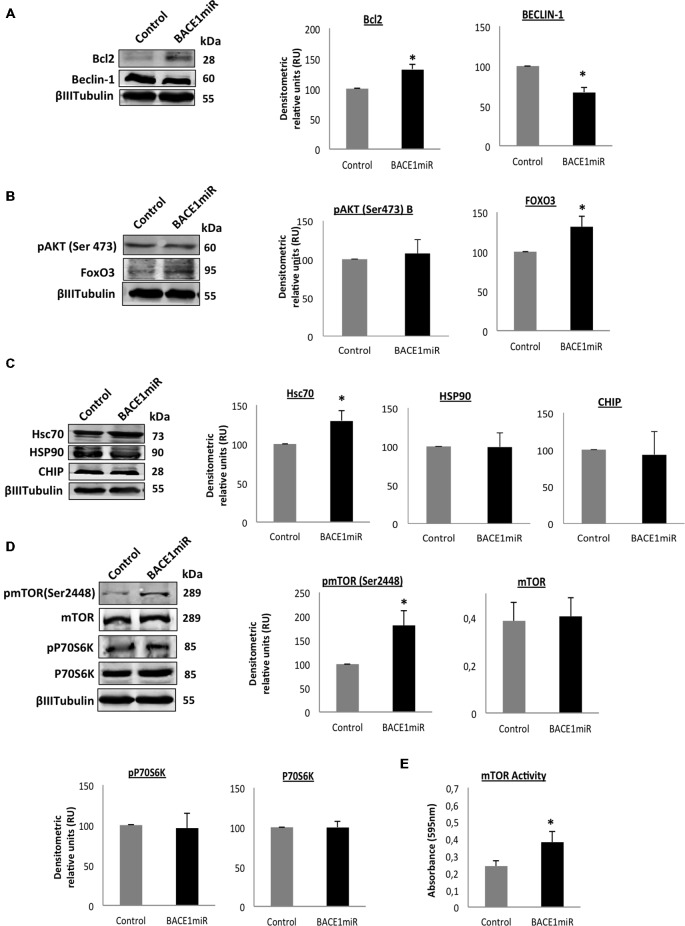
**Silencing of BACE1 upregulates the survival signaling in 3xTg-AD mice.** Survival and quality control regulation pathways were analyzed. **(A)** Bcl2/Beclin-1, **(B)** Akt/FoxO3, **(C)** Hsc70/Hsp90/CHIP protein levels, **(D)** mTOR pathway and its activity **(E)** from the hippocampi of 3xTg-AD mice 24-month-old after 6 months of injection with AAV2/5-BACE1miR and AAV2/5-GFP version as control. Representative blots are shown. RU = relative unit. *n* = 4, *p < 0.05.

We evaluated two of the most important mechanisms of autophagic regulation pathways, Akt/mTOR and Bcl2/Beclin-1 pathways (Figures 8A–D). Although, we did not detect changes of pSer473 AKT in hippocampi total lysates (Figure 8B), we detected high protein levels of p2448 mTOR and increased mTOR activity (Figure 8E) in the hippocampi of 3xTg-AD treated, without changes in pThr389 p70S6K with BACE1miR compared with the control values, (Figures 8C,D). Complementarily, the BACEmiR treatment produced increased protein levels of BCL2 and a reduction of Beclin 1 (Figure 8A), accompained by an increase of FoXO3, Hsc70, without changes in total lysates of Hsp90 and CHIP. These findings together, maybe suggest that the silencing of BACE1 induces survival and cellular homeostasis.

## Discussion

Our data show, for the first time, that BACE1 targeting-induced protection reduces soluble hyperphosphorylated tau, modulating certain autophagy-related proteins in the hippocampi of aged 3xTg-AD mice.

Cholesterol homeostasis is impaired in AD patients’ brains, thereby enhancing “β- and γ-secretase activities and Aβ production in human brains” (Xiong et al., 2008). Cellular cholesterol levels can modulate APP metabolism, and “cholesterol depletion reduces the association between APP and lipid rafts and disrupts the APP–PS1 interaction” (Guardia-Laguarta et al., 2009). “Aberrant cholesterol trafficking is associated with the potentiation of APP processing by BACE1, leading to an overall increase in Aβ levels” (Burns et al., 2003). In an APP-overexpressing mouse model, a cholesterol-rich diet increases Aβ accumulation, accelerates AD-related pathology (Refolo et al., 2000), and correlates with tau hyper-phosphorylation (Ghribi et al., 2006). In the Tg2576 mouse model and in AD-affected human brains, Aβ dimers appear in lipid rafts and ApoE progressively accumulates with aging, thereby facilitating Aβ fibril formation (Kawarabayashi et al., 2004).

In our study, we found that BACE1 and Hsc70 became upregulated in human AD brains and increased in lipid rafts from 3xTg-AD mouse brains. Some studies have described the presence of heat shock proteins (Hsps) in lipid rafts (Uittenbogaard et al., 1998; Triantafilou et al., 2002; Broquet et al., 2003); more recent studies have strongly implicated molecular chaperones in Aβ and tau pathobiology, particularly Hsc70, Hsp70, Hsp90 and CHIP (Perez et al., 1991; Cisse et al., 1993; Dou et al., 2003; Hatakeyama et al., 2004; Petrucelli et al., 2004; Sahara et al., 2005; Zhang et al., 2005, 2008; Dickey et al., 2006, 2007, 2008; Elliott et al., 2007, 2009; Carrettiero et al., 2009). “Chaperones are upregulated not only in cultured neuronal cells overexpressing mutant forms of APP or treated with synthetic Aβ42 but also in the cortex and hippocampus of transgenic mice expressing mutant APP”. This upregulation is suggested to be a protective cellular response against Aβ (Hoshino et al., 2007). Aβ accumulation reduced CHIP levels, and “Aβ-induced tau pathology can be rescued by restoring CHIP levels” (Oddo et al., 2008).

BACE1miR was expected to reduce β-amyloidosis (Luo et al., 2001; Kao et al., 2004; Ohno et al., 2004; Laird et al., 2005) and also reduce the levels of hyperphosphorylated tau. However, it did not involve CDK5, GSK3β and PP2A modulation, but involved MAPK in the reduction of pSer396/pSer404 Tau (PHF-1; Augustinack et al., 2002) and other autophagy mediators in a non-conventional way. Interestingly, we found that the BACE1miR induced the translocation of the chaperone proteins Hsp90 and CHIP, which became drastically decreased in the nucleus and significantly increased in the cytoplasm, and Hsc70 was also increased in the membrane fraction, but maybe in a non transcriptional manner, because HSF-1 protein levels was reduced (Wang et al., 2006b). The “Hsp70/CHIP chaperone system plays an important role in regulating tau turnover and selectively eliminating abnormal tau species. CHIP interacts directly with Hsp70/90, inducing ubiquitination of the microtubule-associated protein tau” (Petrucelli et al., 2004). The Hsc70-CHIP complex selectively ubiquitinates phosphorylated tau (Shimura et al., 2004). CHIP overexpression may antagonize tau accumulation in AD-affected brains (Zhang et al., 2008). Deleting “CHIP results in the accumulation of soluble phospho-tau in the brain and also sequesters insoluble filamentous aggregates and prevents cell death” (Dickey et al., 2006). Hsp90 inhibitors enhance Hsp90/CHIP-mediated phospho-tau degradation by enhancing endogenous chaperone activity, thereby “facilitating reductions in phospho-tau accumulation and selectively targeting the aberrant phospho-tau species associated with neurotoxicity” (Dickey et al., 2007). Elimination of aggregated tau by Hsp90 inhibition has also been confirmed in both *in vitro* and *in vivo* tauopathy models (Luo et al., 2007). However, our data suggest that BACE1miR-induced soluble PHF clearance is proteasome-independent because proteasome inhibitors did not block the effect of BACE1miR on hyperphosphorylated tau levels in primary neuronal cultures.

The “two major protein degradation systems are the proteasome pathway and the autophagy-lysosome pathway”; damage to lysosomal function is a well-recognized event in AD neurodegeneration. Lysosome pathology in AD-affected brains involves changes in macroautophagy and increased APP proteolysis, and it contributes to Aβ deposition (Grbovic et al., 2003; Cataldo et al., 2004a,b; Yu et al., 2004; Nixon et al., 2005; Boland et al., 2008). Our results suggest that BACE1miR modified some autophagy mediators because we found that the protein levels of LC3B and LAMP2 were upregulated. The transcription of autophagy-related genes, such as LC3B and Bnip3, occurs during fasting, and Akt/PKB activation blocks autophagy (Mammucari et al., 2007). Therefore, pSer473 Akt downregulation by BACE1miR could upregulate autophagy-related genes, thereby increasing LC3B. By contrast, we found that LC3B was reduced in the membrane fraction of BACE1miR-treated 3xTg-AD mouse hippocampi. However, a significant increase in Hsc70/LAMP2 in lipid rafts was accompanied by a reduction in hyperphosphorylated tau *in vivo*, which could suggest some repercussion of BACE1miR on CMA.

Similarly, two major CMA proteins, Hsc70 and Hsp90, play critical roles in LAMP-2A complex dynamics on the lysosome membrane (Bandyopadhyay et al., 2008). LAMP-2 is a lysosome protein in mammalian cells, and it is necessary for the fusion of lysosomes with autophagosomes (Tanaka et al., 2000; Bampton et al., 2005). The “lysosomal levels of both Hsc70 and LAMP-2A increase when CMA is activated” (Agarraberes et al., 1997; Cuervo and Dice, 2000b). Changes in the dynamic distribution of LAMP-2A into and out of discrete lysosome membrane microdomains contribute to the regulation of CMA, and the number of Hsc70-containing lysosomes increases in conditions that produce CMA activation (Kaushik et al., 2006). CMA declines with age because of decreased levels of the lysosome-associated membrane protein LAMP-2 and decreased substrate binding to the lysosomes (Cuervo and Dice, 2000a,b; Massey et al., 2006; Kiffin et al., 2007). Interestingly, our findings showed that Hsc70 and LAMP2-A were increased by BACE1miR in lipid rafts and in the cytoplasm of 3xTg-AD mouse brains.

Also, we consistently showed that BACE1miR significantly reduced both Hsp90 and pSer473 AKT *in vitro*. It is well documented that Hsp90 inhibitors (geldanamycin, radicicol, and their analogs) dephosphorylate and inactivate Akt (Fujita et al., 2002), which is associated with both tauopathy and cancer inhibition (Georgescu, 2010; Jimenez et al., 2011; Jinwal et al., 2011; Kannaiyan et al., 2011; Chen et al., 2012). Additionally, downregulation of the HSF-1 transcription factor could decrease Hsp90 and CHIP in the nucleus (Jolly et al., 1997; Ali et al., 1998; Bharadwaj et al., 1999; Zhao et al., 2002; Kim et al., 2005; Wang et al., 2006b) and downregulate pAkt expression (Fujita et al., 2002; Yun and Matts, 2005a,b). Moreover, Hsp90 silencing upregulates Bcl-2 protection against 6-OH DA (Alani et al., 2014). Therefore, it has been suggested that heat shock proteins and autophagy cooperate for quality control (Dokladny et al., 2015). In addition, mTOR activity control misfolding protein in metabolic dyshomeostasis (Qian et al., 2010), how our data suggests in this study.

On the other hand, our findings correlate with BACE1miR-induced neuroprotection, which generated a reduction of sustained tau phosphorylation, and involved heat shock proteins, phosphatidylethanolamine and MAPK in a similar manner that to control of cell stress (Dodson et al., 2013) and cancer inhibition. Phosphatidylethanolamine binding proteins block MAPK activation and thereby have potential therapeutic implications in Alzheimer’s disease and cancer (Al-Mulla et al., 2013; Ling et al., 2014). Additionally, Raf, a component of the MAPK cascade, interacts with Hsc70 in the mouse hippocampus in basal conditions (Bonfiglio et al., 2011; Al-Mulla et al., 2013). However, the exact mechanisms by which BACE1miR could modulate those targets remain unknown and require additional studies for understanding the molecular convergence of these actors and their concomitant actions.

Finally, autophagy inhibitors reduced the BACE1miR-induced LC3B levels *in vitro*, and the BACE1miR-mediated soluble hyperphosphorylated tau downregulation was only blocked by 3-MA (a class III PI3K inhibitor), but not by bafilomycin or NH_4_Cl *in vitro*. Together, these data maybe suggest a role for BACE1miR in the double-membrane vesicles and in membrane fusion to form autophagosomes, which requires phosphatidylethanolamine, but they do not imply a typical autophagic flow because insoluble tau was not reduced, neither by bafilomycin nor NH_4_Cl blocked the reduction of hyperphosphorylated tau. Recently, our unpublished data show the repercussion of shBACE1miR on the cellular homeostasis induced by a favorable change of the fatty acid composition of phospholipids, mainly on LPE and ePE, by reduction of arachidonic acid (20:4), increase of DHA (22:6), reduction of cPLA2 activity, reduction of COX2 levels and improving of the cognitive function after 6 and 12 months post-injection (Villamil-Ortiz and Cardona-Gomez, 2015). Concomitantly, it could be reflected in the reduction of proinflammatory signaling as MAPK activity obtained in this study under the same experimental condition, which could be to favor of the modulation of CMA and macroautophagy proteins, and clearance of hyperphosphorylated tau. Because, MAPK regulates downstream cPLA2 under lipid peroxidation (Shibata et al., 2011); being the cPLA2 encharged of the formation of derivated plasmanogens of PE (Makide et al., 2009), and this enzyme activation is close related to hyperphosphorylated tau, inflammation and neurodegeneration (Sundaram et al., 2012). Therefore, the inhibition of PE lipidation of LC3B by 3MA, could be affecting the action of BACE1 silencing on PE fatty acid composition. However, future studies must be developed to determine the specific mechanism of that regulation by BACE1miR. Also, it has recently been shown that phosphatidylethanolamine regulates autophagy and longevity (Rockenfeller et al., 2015), and LC3 lipidation are involved in autophagy and lipid metabolism turnover (Singh et al., 2009), which could be related with tissue homeostasis and improvement of cognitive function of 3xTg-AD mice treated with BACE1miR during 6 and 12 months (Villamil-Ortiz and Cardona-Gomez, 2015).

In summary, our study has extended the role of BACE1 beyond its role in APP cleavage by linking it to Hsc70 and other mediators of autophagy. BACE1 in lipid rafts is associated with tau aggregation, but its silencing induces cellular homeostasis in PE-dependent mode, produce soluble PHF clearance, involving MAPK inhibition, Hsc70 and LAMP2 in lipid rafts maybe favoring CMA. These results emphasize that BACE1 targeting is a promising neuroprotective therapy for Alzheimer’s disease.

## Author Contributions

DP, “design, acquisition of data, analysis and interpretation of data, write the manuscript”; JFC-A, “design, acquisition of data, analysis and interpretation of data”; RLB, “design, critical revision”; AV-L, “acquision of data”; KSK, “analysis data, critical revision”; JCG-G, “design, acquisition of data, analysis and interpretation of data, critical revision”; GPC-G, “design, acquisition of data, analysis and interpretation of data, write the manuscript”.

## Conflict of Interest Statement

The authors declare that the research was conducted in the absence of any commercial or financial relationships that could be construed as a potential conflict of interest.

## Abbreviations

AD: Alzheimer’s disease
AAV: Adenoassociated viral vectors
BACE1: β-site APP cleaving enzyme 1
BACE1miR: shRNA miR sequences for silencing BACE1
CMA: chaperone-mediated autophagy
Hsc70: heat shock protein
CHIP: Hsc70 interacting protein
MAPK: Microtubule associated protein kinase
PHF: paired helicoidal filaments
3MA: 3 methyladenine
3xTg: triple transgenic.

## References

[B1] Agarraberes F. A. Terlecky S. R. Dice J. F. (1997). An intralysosomal hsp70 is required for a selective pathway of lysosomal protein degradation. J. Cell Biol. 137, 825–834. 10.1083/jcb.137.4.825 9151685 PMC2139836

[B2] Ahmed R. R. Holler C. J. Webb R. L. Li F. Beckett T. L. Murphy M. P. (2010). BACE1 and BACE2 enzymatic activities in Alzheimer’s disease. J. Neurochem. 112, 1045–1053. 10.1111/j.1471-4159.2009.06528.x 19968762 PMC2819564

[B3] Alani B. Salehi R. Sadeghi P. Zare M. Khodagholi F. Arefian E.. (2014). Silencing of Hsp90 chaperone expression protects against 6-hydroxydopamine toxicity in PC12 cells. J. Mol. Neurosci. 52, 392–402. 10.1007/s12031-013-0163-9 24234033

[B4] Ali A. Bharadwaj S. O’Carroll R. Ovsenek N. (1998). HSP90 interacts with and regulates the activity of heat shock factor 1 in Xenopus oocytes. Mol. Cell. Biol. 18, 4949–4960. 10.1128/mcb.18.9.4949 9710578 PMC109079

[B5] Al-Mulla F. Bitar M. S. Thiery J. P. Zea T. T. Chatterjee D. Bennett L.. (2013). Clinical implications for loss or diminution of expression of Raf-1 kinase inhibitory protein and its phosphorylated form in ductal breast cancer. Am. J. Cancer Res. 3, 446–464. 24224123 PMC3816965

[B6] Augustinack J. C. Schneider A. Mandelkow E. M. Hyman B. T. (2002). Specific tau phosphorylation sites correlate with severity of neuronal cytopathology in Alzheimer’s disease. Acta Neuropathol. 103, 26–35. 10.1007/s004010100423 11837744

[B7] Balch W. E. Morimoto R. I. Dillin A. Kelly J. W. (2008). Adapting proteostasis for disease intervention. Science 319, 916–919. 10.1126/science.1141448 18276881

[B8] Ballinger C. A. Connell P. Wu Y. Hu Z. Thompson L. J. Yin L. Y.. (1999). Identification of CHIP, a novel tetratricopeptide repeat-containing protein that interacts with heat shock proteins and negatively regulates chaperone functions. Mol. Cell. Biol. 19, 4535–4545. 10.1128/mcb.19.6.4535 10330192 PMC104411

[B9] Bampton E. T. Goemans C. G. Niranjan D. Mizushima N. Tolkovsky A. M. (2005). The dynamics of autophagy visualized in live cells: from autophagosome formation to fusion with endo/lysosomes. Autophagy 1, 23–36. 10.4161/auto.1.1.1495 16874023

[B10] Bandyopadhyay U. Kaushik S. Varticovski L. Cuervo A. M. (2008). The chaperone-mediated autophagy receptor organizes in dynamic protein complexes at the lysosomal membrane. Mol. Cell. Biol. 28, 5747–5763. 10.1128/MCB.02070-07 18644871 PMC2546938

[B11] Bharadwaj S. Ali A. Ovsenek N. (1999). Multiple components of the HSP90 chaperone complex function in regulation of heat shock factor 1 *In vivo*. Mol. Cell. Biol. 19, 8033–8041. 10.1128/mcb.19.12.8033 10567529 PMC84888

[B12] Boland B. Kumar A. Lee S. Platt F. M. Wegiel J. Yu W. H.. (2008). Autophagy induction and autophagosome clearance in neurons: relationship to autophagic pathology in Alzheimer’s disease. J. Neurosci. 28, 6926–6937. 10.1523/JNEUROSCI.0800-08.2008 18596167 PMC2676733

[B13] Bonfiglio J. J. Maccarrone G. Rewerts C. Holsboer F. Arzt E. Turck C. W.. (2011). Characterization of the B-Raf interactome in mouse hippocampal neuronal cells. J. Proteomics 74, 186–198. 10.1016/j.jprot.2010.10.006 21055488

[B14] Bonini N. M. (2002). Chaperoning brain degeneration. Proc. Natl. Acad. Sci. U S A 4, 16407–16411. 10.1073/pnas.152330499 12149445 PMC139901

[B15] Boudreau R. L. McBride J. L. Martins I. Shen S. Xing Y. Carter B. J.. (2009). Nonallele-specific silencing of mutant and wild-type huntingtin demonstrates therapeutic efficacy in Huntington’s disease mice. Mol. Ther. 17, 1053–1063. 10.1038/mt.2009.17 19240687 PMC2835182

[B16] Broquet A. H. Thomas G. Masliah J. Trugnan G. Bachelet M. (2003). Expression of the molecular chaperone Hsp70 in detergent-resistant microdomains correlates with its membrane delivery and release. J. Biol. Chem. 278, 21601–21606. 10.1074/jbc.m302326200 12682040

[B17] Burns M. Gaynor K. Olm V. Mercken M. LaFrancois J. Wang L.. (2003). Presenilin redistribution associated with aberrant cholesterol transport enhances beta-amyloid production *in vivo*. J. Neurosci. 23, 5645–5649. 12843267 10.1523/JNEUROSCI.23-13-05645.2003 PMC6741250

[B18] Cardona-Gomez P. Perez M. Avila J. Garcia-Segura L. M. Wandosell F. (2004). Estradiol inhibits GSK3 and regulates interaction of estrogen receptors, GSK3 and beta-catenin in the hippocampus. Mol. Cell. Neurosci. 25, 363–373. 10.1016/j.mcn.2003.10.008 15033165

[B19] Carrettiero D. C. Hernandez I. Neveu P. Papagiannakopoulos T. Kosik K. S. (2009). The cochaperone BAG2 sweeps paired helical filament-insoluble tau from the microtubule. J. Neurosci. 29, 2151–2161. 10.1523/JNEUROSCI.4660-08.2009 19228967 PMC2768429

[B190] Castro-Alvarez J. F. Uribe-Arias A. Kosik K. S. Cardona-Gómez G. P. (2014). Long- and short-term CDK5 knockdown prevents spatial memory dysfunction and tau pathology of triple transgenic Alzheimer’s mice. Front. Aging Neurosci. 6:243 10.3389/fnagi.2014.00243 25309427 PMC4159979

[B20] Cataldo A. M. Petanceska S. Terio N. B. Peterhoff C. M. Durham R. Mercken M.. (2004a). Abeta localization in abnormal endosomes: association with earliest Abeta elevations in AD and Down syndrome. Neurobiol. Aging 25, 1263–1272. 10.1016/j.neurobiolaging.2004.02.027 15465622

[B21] Cataldo A. M. Peterhoff C. M. Schmidt S. D. Terio N. B. Duff K. Beard M.. (2004b). Presenilin mutations in familial Alzheimer disease and transgenic mouse models accelerate neuronal lysosomal pathology. J. Neuropathol. Exp. Neurol. 63, 821–830. 15330337 10.1093/jnen/63.8.821

[B23] Chen S. Brown I. R. (2007). Neuronal expression of constitutive heat shock proteins: implications for neurodegenerative diseases. Cell Stress Chaperones 12, 51–58. 10.1379/csc-236r.1 17441507 PMC1852893

[B22] Chen L. M. Xiong Y. S. Kong F. L. Qu M. Wang Q. Chen X. Q.. (2012). Neuroglobin attenuates Alzheimer-like tau hyperphosphorylation by activating Akt signaling. J. Neurochem. 120, 157–164. 10.1111/j.1471-4159.2011.07275.x 21496024

[B24] Cisse S. Perry G. Lacoste-Royal G. Cabana T. Gauvreau D. (1993). Immunochemical identification of ubiquitin and heat-shock proteins in corpora amylacea from normal aged and Alzheimer’s disease brains. Acta Neuropathol. 85, 233–240. 10.1007/bf00227716 7681614

[B25] Cordy J. M. Hussain I. Dingwall C. Hooper N. M. Turner A. J. (2003). Exclusively targeting beta-secretase to lipid rafts by GPI-anchor addition up-regulates beta-site processing of the amyloid precursor protein. Proc. Natl. Acad. Sci. U S A 100, 11735–11740. 10.1073/pnas.1635130100 14504402 PMC208827

[B26] Cuervo A. M. Dice J. F. (2000a). Age-related decline in chaperone-mediated autophagy. J. Biol. Chem. 275, 31505–31513. 10.1074/jbc.m002102200 10806201

[B27] Cuervo A. M. Dice J. F. (2000b). Regulation of lamp2a levels in the lysosomal membrane. Traffic 1, 570–583. 10.1034/j.1600-0854.2000.010707.x 11208145

[B28] Dickey C. A. Kamal A. Lundgren K. Klosak N. Bailey R. M. Dunmore J.. (2007). The high-affinity HSP90-CHIP complex recognizes and selectively degrades phosphorylated tau client proteins. J. Clin. Invest. 117, 648–658. 10.1172/jci29715 17304350 PMC1794119

[B29] Dickey C. A. Koren J. Zhang Y. J. Xu Y. F. Jinwal U. K. Birnbaum M. J.. (2008). Akt and CHIP coregulate tau degradation through coordinated interactions. Proc. Natl. Acad. Sci. U S A 105, 3622–3627. 10.1073/pnas.0709180105 18292230 PMC2265134

[B30] Dickey C. A. Yue M. Lin W. L. Dickson D. W. Dunmore J. H. Lee W. C.. (2006). Deletion of the ubiquitin ligase CHIP leads to the accumulation, but not the aggregation, of both endogenous phospho- and caspase-3-cleaved tau species. J. Neurosci. 26, 6985–6996. 10.1523/jneurosci.0746-06.2006 16807328 PMC6673930

[B31] Dodson M. Darley-Usmar V. Zhang J. (2013). Cellular metabolic and autophagic pathways: traffic control by redox signaling. Free Radic. Biol. Med. 63, 207–221. 10.1016/j.freeradbiomed.2013.05.014 23702245 PMC3729625

[B32] Dokladny K. Myers O. B. Moseley P. L. (2015). Heat shock response and autophagy-cooperation and control. Autophagy 11, 200–213. 10.1080/15548627.2015.1009776 25714619 PMC4502786

[B33] Dou F. Netzer W. J. Tanemura K. Li F. Hartl F. U. Takashima A.. (2003). Chaperones increase association of tau protein with microtubules. Proc. Natl. Acad. Sci. U S A 100, 721–726. 10.1073/pnas.242720499 12522269 PMC141063

[B34] Doyle S. M. Genest O. Wickner S. (2013). Protein rescue from aggregates by powerful molecular chaperone machines. Nat. Rev. Mol. Cell Biol. 14, 617–629. 10.1038/nrm3660 24061228

[B35] D’Souza S. M. Brown I. R. (1998). Constitutive expression of heat shock proteins Hsp90, Hsc70, Hsp70 and Hsp60 in neural and non-neural tissues of the rat during postnatal development. Cell Stress Chaperones 3, 188–199. 10.1379/1466-1268(1998)003<0188:ceohsp>2.3.co;2 9764759 PMC312963

[B36] Ehehalt R. Keller P. Haass C. Thiele C. Simons K. (2003). Amyloidogenic processing of the Alzheimer beta-amyloid precursor protein depends on lipid rafts. J. Cell Biol. 160, 113–123. 10.1083/jcb.200207113 12515826 PMC2172747

[B37] Elliott E. Laufer O. Ginzburg I. (2009). BAG-1M is up-regulated in hippocampus of Alzheimer’s disease patients and associates with tau and APP proteins. J. Neurochem. 109, 1168–1178. 10.1111/j.1471-4159.2009.06047.x 19317853

[B38] Elliott E. Tsvetkov P. Ginzburg I. (2007). BAG-1 associates with Hsc70.Tau complex and regulates the proteasomal degradation of Tau protein. J. Biol. Chem. 282, 37276–37284. 10.1074/jbc.m706379200 17954934

[B39] Fujita N. Sato S. Ishida A. Tsuruo T. (2002). Involvement of Hsp90 in signaling and stability of 3-phosphoinositide-dependent kinase-1. J. Biol. Chem. 277, 10346–10353. 10.1074/jbc.m106736200 11779851

[B40] Fukumoto H. Cheung B. S. Hyman B. T. Irizarry M. C. (2002). Beta-secretase protein and activity are increased in the neocortex in Alzheimer disease. Arch. Neurol. 59, 1381–1389. 10.1001/archneur.59.9.1381 12223024

[B41] Georgescu M. M. (2010). PTEN tumor suppressor network in PI3K-Akt pathway control. Genes Cancer 1, 1170–1177. 10.1177/1947601911407325 21779440 PMC3092286

[B42] Ghribi O. Larsen B. Schrag M. Herman M. M. (2006). High cholesterol content in neurons increases BACE, beta-amyloid and phosphorylated tau levels in rabbit hippocampus. Exp. Neurol. 200, 460–467. 10.1016/j.expneurol.2006.03.019 16696972

[B43] Grbovic O. M. Mathews P. M. Jiang Y. Schmidt S. D. Dinakar R. Summers-Terio N. B.. (2003). Rab5-stimulated up-regulation of the endocytic pathway increases intracellular beta-cleaved amyloid precursor protein carboxyl-terminal fragment levels and Abeta production. J. Biol. Chem. 278, 31261–31268. 10.1074/jbc.m304122200 12761223

[B44] Grimm M. O. Grimm H. S. Tomic I. Beyreuther K. Hartmann T. Bergmann C. (2008). Independent inhibition of Alzheimer disease beta- and gamma-secretase cleavage by lowered cholesterol levels. J. Biol. Chem. 283, 11302–11311. 10.1074/jbc.M801520200 18308724

[B45] Guardia-Laguarta C. Coma M. Pera M. Clarimón J. Sereno L. Agulló J. M.. (2009). Mild cholesterol depletion reduces amyloid-beta production by impairing APP trafficking to the cell surface. J. Neurochem. 110, 220–230. 10.1111/j.1471-4159.2009.06126.x 19457132 PMC2741735

[B450] Gutiérrez-Vargas J. A. Múnera A. Cardona-Gómez G. P. (2015). CDK5 knockdown prevents hippocampal degeneration and cognitive dysfunction produced by cerebral ischemia. J. Cereb. Blood Flow Metab. 35, 1937–1949. 10.1038/jcbfm.2015.150 26104286 PMC4671113

[B46] Hamano T. Gendron T. F. Causevic E. Yen S. H. Lin W. L. Isidoro C.. (2008). Autophagic-lysosomal perturbation enhances tau aggregation in transfectants with induced wild-type tau expression. Eur. J. Neurosci. 27, 1119–1130. 10.1111/j.1460-9568.2008.06084.x 18294209

[B47] Hatakeyama S. Matsumoto M. Kamura T. Murayama M. Chui D. H. Planel E.. (2004). U-box protein carboxyl terminus of Hsc70-interacting protein (CHIP) mediates poly-ubiquitylation preferentially on four-repeat Tau and is involved in neurodegeneration of tauopathy. J. Neurochem. 91, 299–307. 10.1111/j.1471-4159.2004.02713.x 15447663

[B48] Holsinger R. M. Lee J. S. Boyd A. Masters C. L. Collins S. J. (2006). CSF BACE1 activity is increased in CJD and Alzheimer disease versus [corrected] other dementias. Neurology 67, 710–712. 10.1212/01.wnl.0000229925.52203.4c 16924032

[B49] Hoshino T. Nakaya T. Araki W. Suzuki K. Suzuki T. Mizushima T. (2007). Endoplasmic reticulum chaperones inhibit the production of amyloid-beta peptides. Biochem. J. 402, 581–589. 10.1042/bj20061318 17132139 PMC1863563

[B50] Ikeda K. Akiyama H. Arai T. Kondo H. Haga C. Iritani S.. (1998). Alz-50/Gallyas-positive lysosome-like intraneuronal granules in Alzheimer’s disease and control brains. Neurosci. Lett. 258, 113–116. 10.1016/s0304-3940(98)00867-2 9875540

[B51] Jiang J. Ballinger C. A. Wu Y. Dai Q. Cyr D. M. Höhfeld J.. (2001). CHIP is a U-box-dependent E3 ubiquitin ligase: identification of Hsc70 as a target for ubiquitylation. J. Biol. Chem. 276, 42938–42944. 10.1074/jbc.m101968200 11557750

[B52] Jimenez S. Torres M. Vizuete M. Sanchez-Varo R. Sanchez-Mejias E. Trujillo-Estrada L.. (2011). Age-dependent accumulation of soluble amyloid beta (Abeta) oligomers reverses the neuroprotective effect of soluble amyloid precursor Protein-α (sAPPα) by modulating phosphatidylinositol 3-kinase (PI3K)/Akt-GSK-3beta pathway in Alzheimer mouse model. J. Biol. Chem. 286, 18414–18425. 10.1074/jbc.M110.209718 21460223 PMC3099658

[B53] Jinwal U. K. Trotter J. H. Abisambra J. F. Koren J. IIIrd Lawson L. Y. Vestal G. D.. (2011). The Hsp90 kinase co-chaperone Cdc37 regulates tau stability and phosphorylation dynamics. J. Biol. Chem. 286, 16976–16983. 10.1074/jbc.M110.182493 21367866 PMC3089541

[B54] Jolly C. Morimoto R. Robert-Nicoud M. Vourc’h C. (1997). HSF1 transcription factor concentrates in nuclear foci during heat shock: relationship with transcription sites. J. Cell Sci. 110, 2935–2941. 9359877 10.1242/jcs.110.23.2935

[B55] Kalvodova L. Kahya N. Schwille P. Ehehalt R. Verkade P. Drechsel D.. (2005). Lipids as modulators of proteolytic activity of BACE: involvement of cholesterol, glycosphingolipids and anionic phospholipids *in vitro*. J. Biol. Chem. 280, 36815–36823. 10.1074/jbc.m504484200 16115865

[B56] Kampinga H. H. Hageman J. Vos M. J. Kubota H. Tanguay R. M. Bruford E. A.. (2009). Guidelines for the nomenclature of the human heat shock proteins. Cell Stress Chaperones 14, 105–111. 10.1007/s12192-008-0068-7 18663603 PMC2673902

[B57] Kannaiyan R. Manu K. A. Chen L. Li F. Rajendran P. Subramaniam A.. (2011). Celastrol inhibits tumor cell proliferation and promotes apoptosis through the activation of c-Jun N-terminal kinase and suppression of PI3 K/Akt signaling pathways. Apoptosis 16, 1028–1041. 10.1007/s10495-011-0629-6 21786165

[B58] Kao S. C. Krichevsky A. M. Kosik K. S. Tsai L. H. (2004). BACE1 suppression by RNA interference in primary cortical neurons. J. Biol. Chem. 279, 1942–1949. 10.1074/jbc.m309219200 14600149

[B59] Karran E. Mercken M. De Strooper B. (2011). The amyloid cascade hypothesis for Alzheimer’s disease: an appraisal for the development of therapeutics. Nat. Rev. Drug Discov. 10, 698–712. 10.1038/nrd3505 21852788

[B60] Kaushik S. Massey A. C. Cuervo A. M. (2006). Lysosome membrane lipid microdomains: novel regulators of chaperone-mediated autophagy. EMBO J. 25, 3921–3933. 10.1038/sj.emboj.7601283 16917501 PMC1560360

[B61] Kawarabayashi T. Shoji M. Younkin L. H. Wen-Lang L. Dickson D. W. Murakami T.. (2004). Dimeric amyloid beta protein rapidly accumulates in lipid rafts followed by apolipoprotein E and phosphorylated tau accumulation in the Tg2576 mouse model of Alzheimer’s disease. J. Neurosci. 24, 3801–3809. 10.1523/jneurosci.5543-03.2004 15084661 PMC6729359

[B62] Kiffin R. Kaushik S. Zeng M. Bandyopadhyay U. Zhang C. Massey A. C.. (2007). Altered dynamics of the lysosomal receptor for chaperone-mediated autophagy with age. J. Cell Sci. 120, 782–791. 10.1242/jcs.001073 17284523

[B63] Kim S. A. Yoon J. H. Kim D. K. Kim S. G. Ahn S. G. (2005). CHIP interacts with heat shock factor 1 during heat stress. FEBS Lett. 579, 6559–6563. 10.1016/j.febslet.2005.10.043 16293251

[B64] Kins S. Lauther N. Szodorai A. Beyreuther K. (2006). Subcellular trafficking of the amyloid precursor protein gene family and its pathogenic role in Alzheimer’s disease. Neurodegener. Dis. 3, 218–226. 10.1159/000095259 17047360

[B65] Kumar P. Ambasta R. K. Veereshwarayya V. Rosen K. M. Kosik K. S. Band H.. (2007). CHIP and HSPs interact with beta-APP in a proteasome-dependent manner and influence Abeta metabolism. Hum. Mol. Genet. 16, 848–864. 10.1093/hmg/ddm030 17317785

[B66] Laird F. M. Cai H. Savonenko A. V. Farah M. H. He K. Melnikova T.. (2005). BACE1, a major determinant of selective vulnerability of the brain to amyloid-beta amyloidogenesis, is essential for cognitive, emotional and synaptic functions. J. Neurosci. 25, 11693–11709. 10.1523/jneurosci.2766-05.2005 16354928 PMC2564291

[B67] Lee K. S. Chung J. H. Oh B. H. Hong C. H. (2008). Increased plasma levels of heat shock protein 70 in patients with vascular mild cognitive impairment. Neurosci. Lett. 436, 223–226. 10.1016/j.neulet.2008.03.025 18394800

[B68] Ling H. H. Mendoza-Viveros L. Mehta N. Cheng H. Y. (2014). Raf kinase inhibitory protein (RKIP): functional pleiotropy in the mammalian brain. Crit. Rev. Oncog. 19, 505–516. 10.1615/critrevoncog.2014011899 25597360 PMC4767416

[B70] Luo Y. Bolon B. Kahn S. Bennett B. D. Babu-Khan S. Denis P.. (2001). Mice deficient in BACE1, the Alzheimer’s beta-secretase, have normal phenotype and abolished beta-amyloid generation. Nat. Neurosci. 4, 231–232. 10.1038/85059 11224535

[B69] Luo W. Dou F. Rodina A. Chip S. Kim J. Zhao Q.. (2007). Roles of heat-shock protein 90 in maintaining and facilitating the neurodegenerative phenotype in tauopathies. Proc. Natl. Acad. Sci. U S A 104, 9511–9516. 10.1073/pnas.0701055104 17517623 PMC1890525

[B71] Makide K. Kitamura H. Sato Y. Okutani M. Aoki J. (2009). Emerging lysophospholipid mediators, lysophosphatidylserine, lysophosphatidylthreonine, lysophosphatidylethanolamine and lysophosphatidylglycerol. Prostaglandins Other Lipid Mediat. 89, 135–139. 10.1016/j.prostaglandins.2009.04.009 19427394

[B72] Mammucari C. Milan G. Romanello V. Masiero E. Rudolf R. Del Piccolo P.. (2007). FoxO3 controls autophagy in skeletal muscle *in vivo*. Cell Metab. 6, 458–471. 10.1016/j.cmet.2007.11.001 18054315

[B73] Massey A. C. Kaushik S. Sovak G. Kiffin R. Cuervo A. M. (2006). Consequences of the selective blockage of chaperone-mediated autophagy. Proc. Natl. Acad. Sci. U S A 103, 5805–5810. 10.1073/pnas.0507436103 16585521 PMC1458654

[B74] Murakami N. Oyama F. Gu Y. McLennan I. S. Nonaka I. Ihara Y. (1998). Accumulation of tau in autophagic vacuoles in chloroquine myopathy. J. Neuropathol. Exp. Neurol. 57, 664–673. 10.1097/00005072-199807000-00003 9690670

[B75] Nixon R. A. Wegiel J. Kumar A. Yu W. H. Peterhoff C. Cataldo A.. (2005). Extensive involvement of autophagy in Alzheimer disease: an immuno-electron microscopy study. J. Neuropathol. Exp. Neurol. 64, 113–122. 15751225 10.1093/jnen/64.2.113

[B76] Oda A. Tamaoka A. Araki W. (2010). Oxidative stress up-regulates presenilin 1 in lipid rafts in neuronal cells. J. Neurosci. Res. 88, 1137–1145. 10.1002/jnr.22271 19885829

[B77] Oddo S. Caccamo A. Shepherd J. D. Murphy M. P. Golde T. E. Kayed R.. (2003). Triple-transgenic model of Alzheimer’s disease with plaques and tangles: intracellular Abeta and synaptic dysfunction. Neuron 39, 409–421. 10.1016/s0896-6273(03)00434-3 12895417

[B78] Oddo S. Caccamo A. Tseng B. Cheng D. Vasilevko V. Cribbs D. H.. (2008). Blocking Abeta42 accumulation delays the onset and progression of tau pathology via the C terminus of heat shock protein70-interacting protein: a mechanistic link between Abeta and tau pathology. J. Neurosci. 28, 12163–12175. 10.1523/JNEUROSCI.2464-08.2008 19020010 PMC6671718

[B79] Ohno M. Sametsky E. A. Younkin L. H. Oakley H. Younkin S. G. Citron M.. (2004). BACE1 deficiency rescues memory deficits and cholinergic dysfunction in a mouse model of Alzheimer’s disease. Neuron 41, 27–33. 10.1016/s0896-6273(03)00810-9 14715132

[B80] Oyama F. Murakami N. Ihara Y. (1998). Chloroquine myopathy suggests that tau is degraded in lysosomes: implication for the formation of paired helical filaments in Alzheimer’s disease. Neurosci. Res. 31, 1–8. 10.1016/s0168-0102(98)00020-0 9704973

[B81] Paz Gavilan M. Vela J. Castaño A. Ramos B. del Río J. C. Vitorica J.. (2006). Cellular environment facilitates protein accumulation in aged rat hippocampus. Neurobiol. Aging 27, 973–982. 10.1016/j.neurobiolaging.2005.05.010 15964666

[B82] Perez N. Sugar J. Charya S. Johnson G. Merril C. Bierer L.. (1991). Increased synthesis and accumulation of heat shock 70 proteins in Alzheimer’s disease. Brain Res. Mol. Brain Res. 11, 249–254. 10.1016/0169-328x(91)90033-t 1661822

[B83] Petrucelli L. Dickson D. Kehoe K. Taylor J. Snyder H. Grover A.. (2004). CHIP and Hsp70 regulate tau ubiquitination, degradation and aggregation. Hum. Mol. Genet. 13, 703–714. 10.1093/hmg/ddh083 14962978

[B830] Piedrahita D. Hernández I. López-Tobón A. Fedorov D. Obara B. Manjunath B. S. (2010). Silencing of CDK5 reduces neurofibrillary tangles in transgenic Alzheimer’s mice. J. Neurosci. 30, 13966–13976. 10.1523/JNEUROSCI.3637-10.2010 20962218 PMC3003593

[B84] Puglielli L. Ellis B. C. Saunders A. J. Kovacs D. M. (2003). Ceramide stabilizes beta-site amyloid precursor protein-cleaving enzyme 1 and promotes amyloid beta-peptide biogenesis. J. Biol. Chem. 278, 19777–19783. 10.1074/jbc.m300466200 12649271

[B85] Qian S. B. Zhang X. Sun J. Bennink J. R. Yewdell J. W. Patterson C. (2010). mTORC1 links protein quality and quantity control by sensing chaperone availability. J. Biol. Chem. 285, 27385–27395. 10.1074/jbc.M110.120295 20605781 PMC2930736

[B86] Reed B. Villeneuve S. Mack W. DeCarli C. Chui H. C. Jagust W. (2014). Associations between serum cholesterol levels and cerebral amyloidosis. JAMA Neurol. 71, 195–200. 10.1001/jamaneurol.2013.5390 24378418 PMC4083819

[B87] Refolo L. M. Malester B. LaFrancois J. Bryant-Thomas T. Wang R. Tint G. S.. (2000). Hypercholesterolemia accelerates the Alzheimer’s amyloid pathology in a transgenic mouse model. Neurobiol. Dis. 7, 321–331. 10.1006/nbdi.2000.0304 10964604

[B88] Refolo L. M. Pappolla M. A. LaFrancois J. Malester B. Schmidt S. D. Thomas-Bryant T.. (2001). A cholesterol-lowering drug reduces beta-amyloid pathology in a transgenic mouse model of Alzheimer’s disease. Neurobiol. Dis. 8, 890–899. 10.1006/nbdi.2001.0422 11592856

[B89] Riddell D. R. Christie G. Hussain I. Dingwall C. (2001). Compartmentalization of beta-secretase (Asp2) into low-buoyant density, noncaveolar lipid rafts. Curr. Biol. 11, 1288–1293. 10.1016/s0960-9822(01)00394-3 11525745

[B90] Rockenfeller P. Koska M. Pietrocola F. Minois N. Knittelfelder O. Sica V.. (2015). Phosphatidylethanolamine positively regulates autophagy and longevity. Cell Death Differ. 22, 499–508. 10.1038/cdd.2014.219 25571976 PMC4326582

[B91] Sahara N. Murayama M. Mizoroki T. Urushitani M. Imai Y. Takahashi R.. (2005). *In vivo* evidence of CHIP up-regulation attenuating tau aggregation. J. Neurochem. 94, 1254–1263. 10.1111/j.1471-4159.2005.03272.x 16111477

[B92] Sakahira H. Breuer P. Hayer-Hartl M. K. Hartl F. U. (2002). Molecular chaperones as modulators of polyglutamine protein aggregation and toxicity. Proc. Natl. Acad. Sci. U S A 4(Suppl. 99), 16412–16418. 10.1073/pnas.182426899 12189209 PMC139902

[B93] Shibata N. Kato Y. Inose Y. Hiroi A. Yamamoto T. Morikawa S.. (2011). 4-Hydroxy-2-nonenal upregulates and phosphorylates cytosolic phospholipase A(2). in cultured Ra2 microglial cells via MAPK pathways. Neuropathology 31, 122–128. 10.1111/j.1440-1789.2010.01139.x 20667012

[B94] Shimura H. Schwartz D. Gygi S. P. Kosik K. S. (2004). CHIP-Hsc70 complex ubiquitinates phosphorylated tau and enhances cell survival. J. Biol. Chem. 279, 4869–4876. 10.1074/jbc.m305838200 14612456

[B95] Singh R. Cuervo A. M. (2011). Autophagy in the cellular energetic balance. Cell Metab. 13, 495–504. 10.1016/j.cmet.2011.04.004 21531332 PMC3099265

[B96] Singh R. Kaushik S. Wang Y. Xiang Y. Novak I. Komatsu M.. (2009). Autophagy regulates lipid metabolism. Nature 458, 1131–1135. 10.1038/nature07976 19339967 PMC2676208

[B97] Sundaram J. R. Chan E. S. Poore C. P. Pareek T. K. Cheong W. F. Shui G.. (2012). Cdk5/p25-induced cytosolic PLA2-mediated lysophosphatidylcholine production regulates neuroinflammation and triggers neurodegeneration. J. Neurosci. 32, 1020–1034. 10.1523/JNEUROSCI.5177-11.2012 22262900 PMC6621136

[B98] Tamagno E. Bardini P. Obbili A. Vitali A. Borghi R. Zaccheo D.. (2002). Oxidative stress increases expression and activity of BACE in NT2 neurons. Neurobiol. Dis. 10, 279–288. 10.1006/nbdi.2002.0515 12270690

[B99] Tamagno E. Guglielmotto M. Aragno M. Borghi R. Autelli R. Giliberto L.. (2008). Oxidative stress activates a positive feedback between the gamma- and beta-secretase cleavages of the beta-amyloid precursor protein. J. Neurochem. 104, 683–695. 18005001 10.1111/j.1471-4159.2007.05072.x PMC2220052

[B100] Tamagno E. Parola M. Bardini P. Piccini A. Borghi R. Guglielmotto M.. (2005). Beta-site APP cleaving enzyme up-regulation induced by 4-hydroxynonenal is mediated by stress-activated protein kinases pathways. J. Neurochem. 92, 628–636. 10.1111/j.1471-4159.2004.02895.x 15659232

[B101] Tanaka Y. Guhde G. Suter A. Eskelinen E. L. Hartmann D. Lüllmann-Rauch R.. (2000). Accumulation of autophagic vacuoles and cardiomyopathy in LAMP-2-deficient mice. Nature 406, 902–906. 10.1038/35022595 10972293

[B102] Tong Y. Zhou W. Fung V. Christensen M. A. Qing H. Sun X.. (2005). Oxidative stress potentiates BACE1 gene expression and Abeta generation. J. Neural Transm. (Vienna) 112, 455–469. 10.1007/s00702-004-0255-3 15614428

[B103] Toyn J. H. Ahlijanian M. K. (2014). Interpreting Alzheimer’s disease clinical trials in light of the effects on amyloid-beta. Alzheimers Res. Ther. 6:14. 10.1186/alzrt244 25031632 PMC4014014

[B104] Triantafilou M. Miyake K. Golenbock D. T. Triantafilou K. (2002). Mediators of innate immune recognition of bacteria concentrate in lipid rafts and facilitate lipopolysaccharide-induced cell activation. J. Cell Sci. 115, 2603–2611. 12045230 10.1242/jcs.115.12.2603

[B105] Tun H. Marlow L. Pinnix I. Kinsey R. Sambamurti K. (2002). Lipid rafts play an important role in A beta biogenesis by regulating the beta-secretase pathway. J. Mol. Neurosci. 19, 31–35. 10.1007/s12031-002-0007-5 12212790

[B106] Uittenbogaard A. Ying Y. Smart E. J. (1998). Characterization of a cytosolic heat-shock protein-caveolin chaperone complex. Involvement in cholesterol trafficking. J. Biol. Chem. 273, 6525–6532. 10.1074/jbc.273.11.6525 9497388

[B107] Urabe M. Ding C. Kotin R. M. (2002). Insect cells as a factory to produce adeno-associated virus type 2 vectors. Hum. Gene Ther. 13, 1935–1943. 10.1089/10430340260355347 12427305

[B108] Vassar R. Kovacs D. M. Yan R. Wong P. C. (2009). The β-secretase enzyme BACE in health and Alzheimer’s disease: regulation, cell biology, function and therapeutic potential. J. Neurosci. 29, 12787–12794. 10.1523/JNEUROSCI.3657-09.2009 19828790 PMC2879048

[B109] Vetrivel K. S. Thinakaran G. (2006). Amyloidogenic processing of beta-amyloid precursor protein in intracellular compartments. Neurology 66, S69–S73. 10.1212/01.wnl.0000192107.17175.39 16432149

[B110] Villamil-Ortiz J. G. Cardona-Gomez G. P. (2015). Comparative analysis of autophagy and tauopathy related markers in cerebral ischemia and Alzheimer’s disease animal models. Front. Aging Neurosci. 7:84. 10.3389/fnagi.2015.00084 26042033 PMC4436888

[B111] Wang Q. J. Ding Y. Kohtz D. S. Mizushima N. Cristea I. M. Rout M. P.. (2006a). Induction of autophagy in axonal dystrophy and degeneration. J. Neurosci. 26, 8057–8068. 10.1523/jneurosci.2261-06.2006 16885219 PMC6673783

[B112] Wang X. Khaleque M. A. Zhao M. J. Zhong R. Gaestel M. Calderwood S. K. (2006b). Phosphorylation of HSF1 by MAPK-activated protein kinase 2 on serine 121, inhibits transcriptional activity and promotes HSP90 binding. J. Biol. Chem. 281, 782–791. 10.1074/jbc.m505822200 16278218

[B113] Wang Y. Martinez-Vicente M. Krüger U. Kaushik S. Wong E. Mandelkow E. M.. (2009). Tau fragmentation, aggregation and clearance: the dual role of lysosomal processing. Hum. Mol. Genet. 18, 4153–4170. 10.1093/hmg/ddp367 19654187 PMC2758146

[B114] Xiong H. Callaghan D. Jones A. Walker D. G. Lue L. F. Beach T. G.. (2008). Cholesterol retention in Alzheimer’s brain is responsible for high beta- and gamma-secretase activities and Abeta production. Neurobiol. Dis. 29, 422–437. 10.1016/j.nbd.2007.10.005 18086530 PMC2720683

[B115] Yang L. B. Lindholm K. Yan R. Citron M. Xia W. Yang X. L.. (2003). Elevated beta-secretase expression and enzymatic activity detected in sporadic Alzheimer disease. Nat. Med. 9, 3–4. 10.1038/nm0103-3 12514700

[B116] Yu W. H. Cuervo A. M. Kumar A. Peterhoff C. M. Schmidt S. D. Lee J. H.. (2005). Macroautophagy–a novel Beta-amyloid peptide-generating pathway activated in Alzheimer’s disease. J. Cell Biol. 171, 87–98. 10.1083/jcb.200505082 16203860 PMC2171227

[B117] Yu W. H. Kumar A. Peterhoff C. Shapiro Kulnane L. Uchiyama Y. Lamb B. T.. (2004). Autophagic vacuoles are enriched in amyloid precursor protein-secretase activities: implications for beta-amyloid peptide over-production and localization in Alzheimer’s disease. Int. J. Biochem. Cell Biol. 36, 2531–2540. 10.1016/j.biocel.2004.05.010 15325590

[B118] Yun B. G. Matts R. L. (2005a). Differential effects of Hsp90 inhibition on protein kinases regulating signal transduction pathways required for myoblast differentiation. Exp. Cell Res. 307, 212–223. 10.1016/j.yexcr.2005.03.003 15922741

[B119] Yun B. G. Matts R. L. (2005b). Hsp90 functions to balance the phosphorylation state of Akt during C2C12 myoblast differentiation. Cell. Signal. 17, 1477–1485. 10.1016/j.cellsig.2005.03.006 15935620

[B120] Zhang J. Y. Liu S. J. Li H. L. Wang J. Z. (2005). Microtubule-associated protein tau is a substrate of ATP/Mg(2+)-dependent proteasome protease system. J. Neural Transm. (Vienna) 112, 547–555. 10.1007/s00702-004-0196-x 15372326

[B121] Zhang Y. J. Xu Y. F. Liu X. H. Li D. Yin J. Liu Y. H.. (2008). Carboxyl terminus of heat-shock cognate 70-interacting protein degrades tau regardless its phosphorylation status without affecting the spatial memory of the rats. J. Neural Transm. (Vienna) 115, 483–491. 10.1007/s00702-007-0857-7 18301957

[B122] Zhao C. Hashiguchi A. Kondoh K. Du W. Hata J. Yamada T. (2002). Exogenous expression of heat shock protein 90 kDa retards the cell cycle and impairs the heat shock response. Exp. Cell Res. 275, 200–214. 10.1006/excr.2002.5501 11969290

